# Supplementary material for: Genomic evidence for rediploidization and adaptive evolution following the whole-genome triplication
Source: Nat Commun. 2024 Feb 22;15:1635. doi: 10.1038/s41467-024-46080-7 (PMC10884412; doi:10.1038/s41467-024-46080-7)
Supplement: Supplementary file 1 — Supplementary Information [file 41467_2024_46080_MOESM1_ESM.pdf]

Genomic evidence for rediploidization and adaptive evolution following the whole-genome triplication

Feng *et al*.

Supplementary Note 1. Genome assembly and quality assessment

Sonneratia alba inhabits low intertidal zones of downstream estuarine systems and is one of the most pervasive and salt-tolerant mangrove species widespread in the Indo West Pacific (IWP) region. Evolving specialized structures such as pneumatophores, *S. alba* demonstrates its waterlogging and salt tolerance, particularly in low intertidal zones. In our previous study, we sequenced the genome of *S. alba* with the N50 value of 5.52 Mb to investigate convergent adaptation in mangrove species[1]. Here, we utilized the three-dimensional proximity information obtained by chromosome conformation capture sequencing (Hi-C) to improve the genome assembly to a chromosome-scale upon the previous version. A total of 103.88 Gb of Hi-C data were generated using the BGISEQ-500 platform for *S. alba* (Supplementary Table 1). Assembled contigs were then clustered into 12 pseudo-chromosomes and 40 unanchored scaffolds. The N50 value was increased from 5.52 Mb to 15.69 Mb (Table 1).

Lagerstroemia speciosa, the closely related inland woody plant, demonstrates adaptability across diverse habitats, including lowland rainforests, riparian areas, as well as urban and rural environments. The cultivation of *L. speciosa* in gardens and urban landscapes highlights its ornamental value and widespread popularity. We assembled the genome of *L. speciosa* by incorporating high-depth PacBio Single-Molecule Real-Time (SMRT) sequencing, Illumina short reads sequencing, and Hi-C technologies. We used a k-mer analysis to estimate genome size at ~340 Mb, consistent with ~361 Mb obtained by flow cytometry. We first generated 95.60 Gb (~299X coverage) of SMRT long reads and 41.16 Gb (~128 coverage) of Illumina short reads (Supplementary Table 1). After preliminary assembly, correction, and polishing, the resulting genome assembly was 319.60 Mb, consistent with the estimated genome size. The longest contig was 8.67 Mb and N50 was 4.72 Mb. The GC content of the assembly was 40.40% (Supplementary Table 10). We then used Hi-C technology to improve the genome assembly. A total of 54.19 Gb of Hi-C data were generated by the Illumina NovaSeq 6000 platform for *L. speciosa* (Supplementary Table 1). Assembled contigs were then clustered into 24 pseudo-chromosomes and 605 unanchored scaffolds. The longest scaffold and N50 were increased to 17.34 Mb and 12.74 Mb (Table 1), respectively.

We examined the integrity of the new assembly of *L. speciosa* by aligning Illumina short reads to the assembly using BWA[2] and CLR subreads using minimap2[3]. In total, 86.83% of CLR subreads and 96.23% of short reads were successfully mapped back to the reference genome. Based on the short reads mapping, we estimated heterozygosity at 11.112 sites per Kb in the *L. speciosa* genome, much higher than 0.226 sites per Kb in *S. alba*. The high heterozygosity in *L. speciosa* was consistent with the k-mer distribution (Supplementary Fig. 30). To assess the completeness of two genome assemblies, we performed BUSCO analysis with the eudicotyledons_odb10 lineage dataset[4]. The BUSCO result

showed that 96.6% and 96.1% more of the 2121 expected plant genes were identified as complete genes, while 2.4% and 2.6% fewer of the genes were considered missing from the assembled genomes of *S. alba* and *L. speciosa* (Supplementary Table 2), respectively. These results suggested that our two genome assemblies matching the chromosome counts recorded in cytological studies[5,6] were of high contiguity, integrity, and accuracy.

Supplementary Note 2. Functional analysis among retained genes in *S. alba* and *L. speciosa*

S. alba and *L. speciosa* had undergone a whole-genome triplication (WGT) event before they diverged from a common ancestor. Following the WGT event, duplicates are typically rapidly lost and retained duplicates provide important sources of evolutionary innovation and aid survival in the newly acquired habitat. For each species, we classified the WGT retentions into different-copy groups (Supplementary Figs 12 and13). We also identified single-copy genes using the duplicate_gene_classifier module from MCScanX[7]. We performed GO enrichment analysis of two-copy and three-copy retention groups after the WGT event with single genes as a control using BiNGO in Cytoscape (v.3.7.2)[8], respectively. We found that most of the retentions were involved in metabolic process, ion binding, and catalytic activity GO categories (Supplementary Figs 27 and 32). Moreover, three-copy groups in *L. speciosa* were also enriched in the cellular component, like the ribosome, extracellular matrix, and external encapsulating structure. For both *S. alba* and *L. speciosa*, the preferential retention groups of transcription regulation and energy metabolism pertained to adaptation to a new environment.

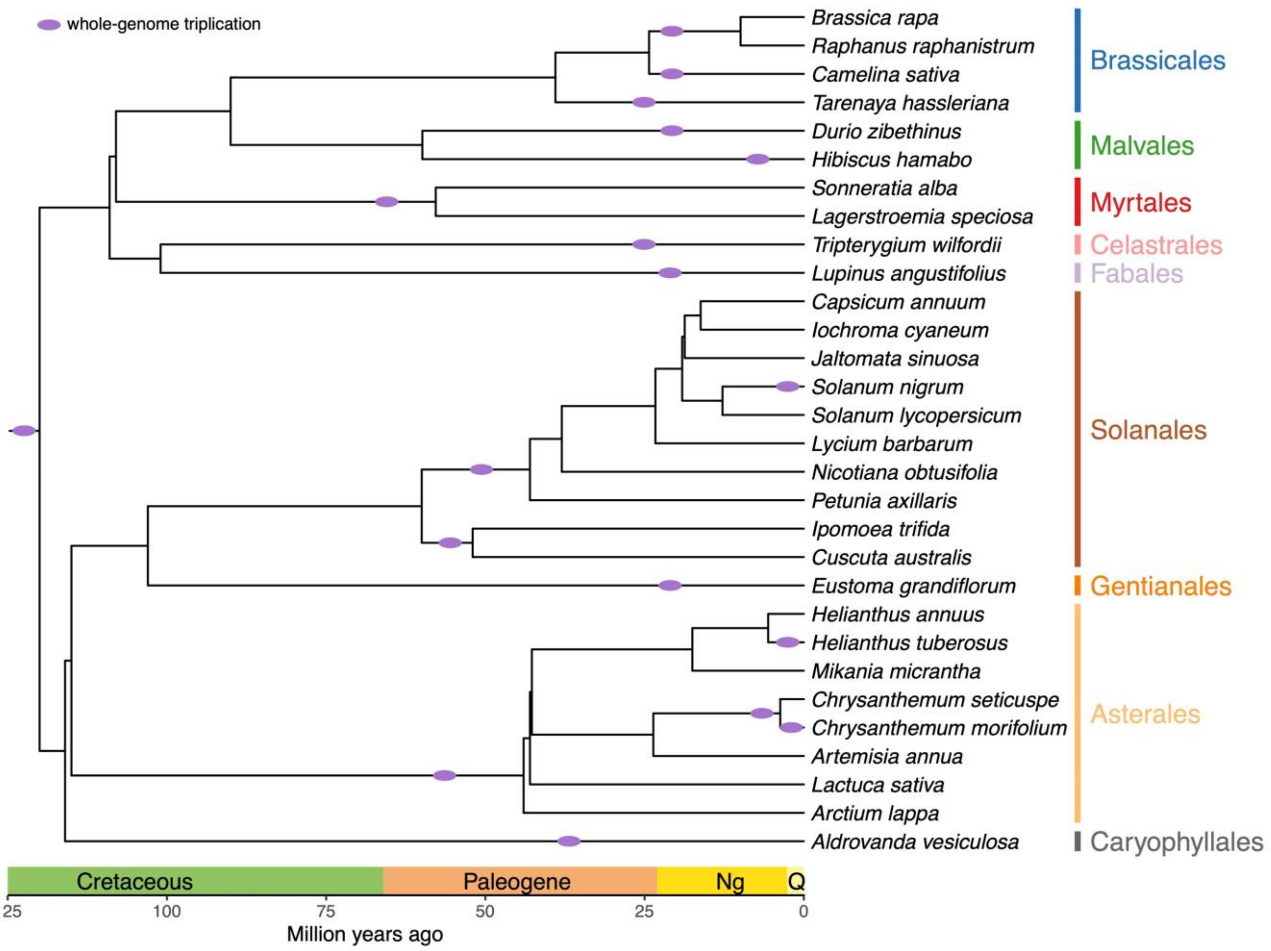

Supplementary Figure 1. Whole-genome triplication events among eudicots.

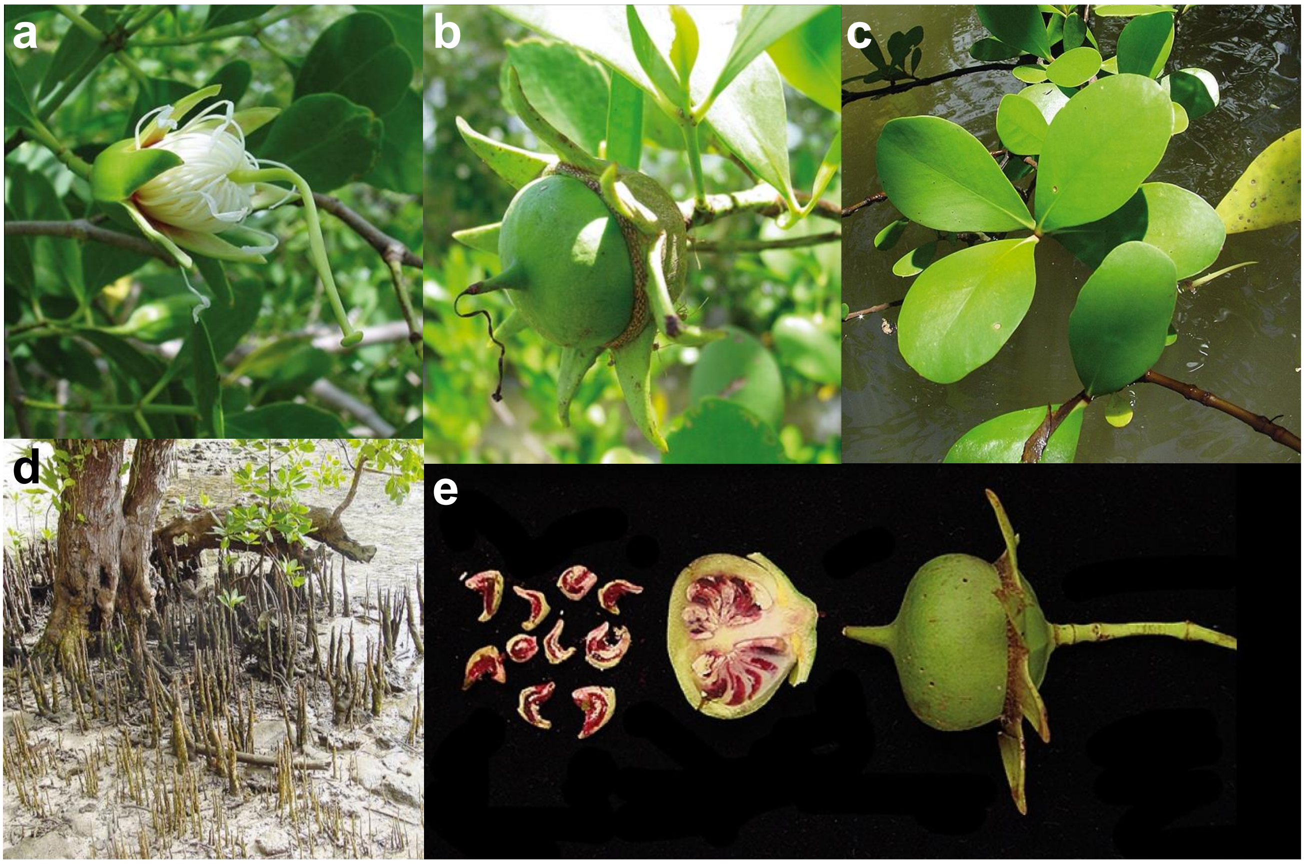

Supplementary Figure 2. Morphology of *Sonneratia alba*. (a) Flowers (b) Fruits (c) Leaves (d) Roots (e) Sectioned fruits and seeds. (a-e from World Mangrove ID e-book contributed by Norman Duke).

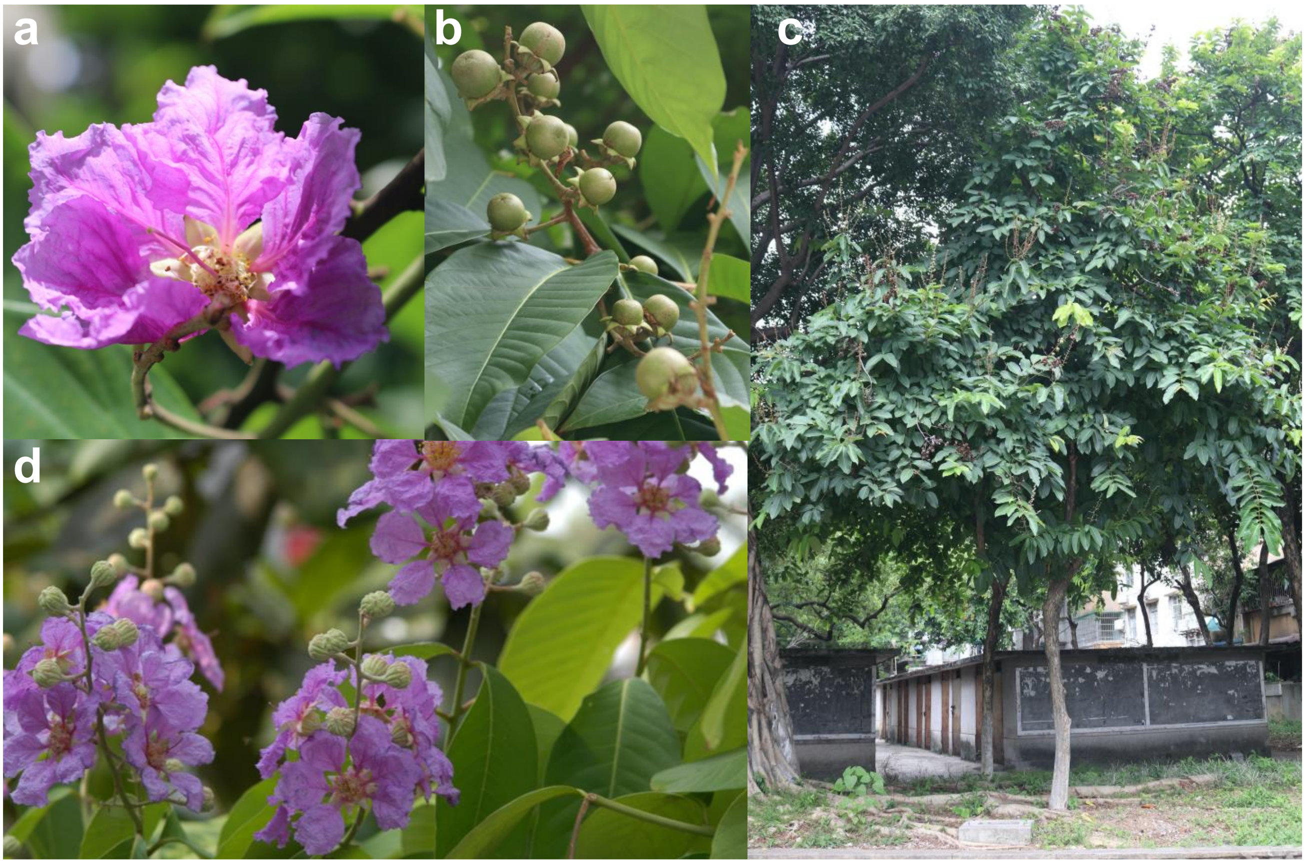

Supplementary Figure 3. Morphology of *Lagerstroemia speciosa*. (a) Flowers (b) Fruits (c) Mature trees (d) Flowers and stellate-tomentose buds. (a-c taken by the author, Xiao Feng, d from Useful Tropical Plants Database contributed by Meneerke bloem).

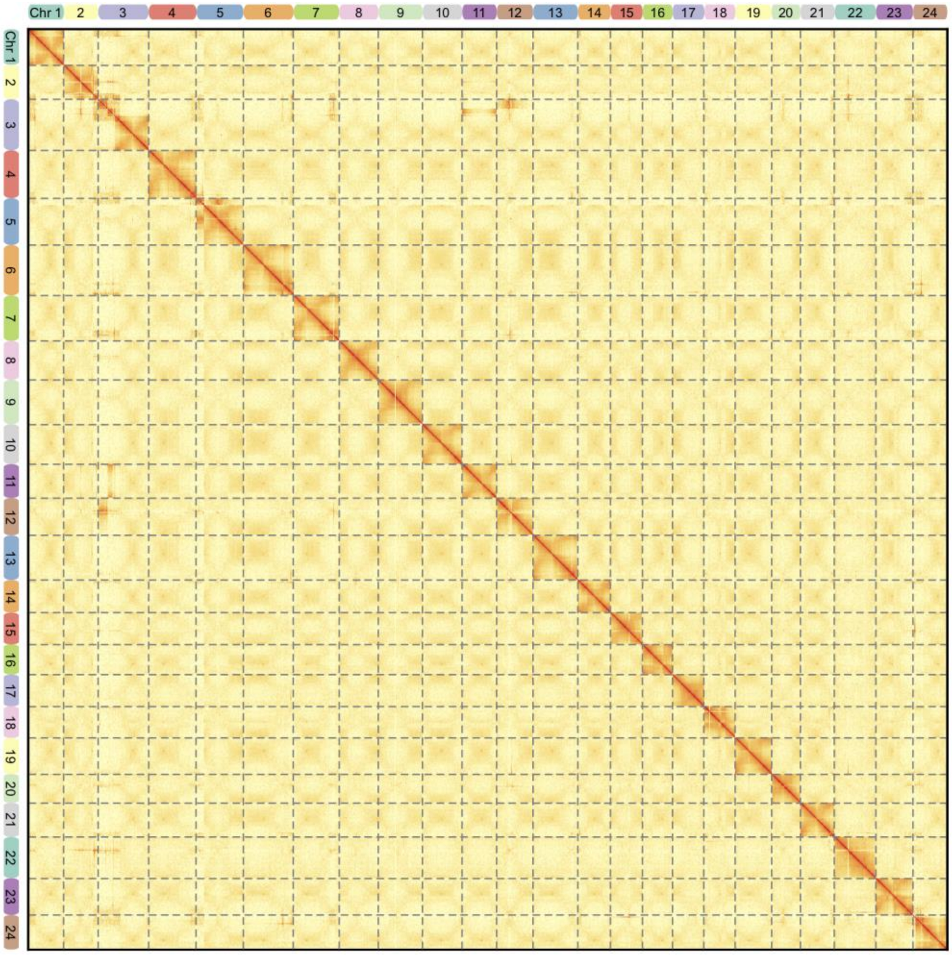

Supplementary Figure 4. Hi-C interactive heatmap of the genome-wide organization of *Lagerstroemia speciosa*. The deeper red means the stronger interaction between the DNA regions. Chr: chromosome.

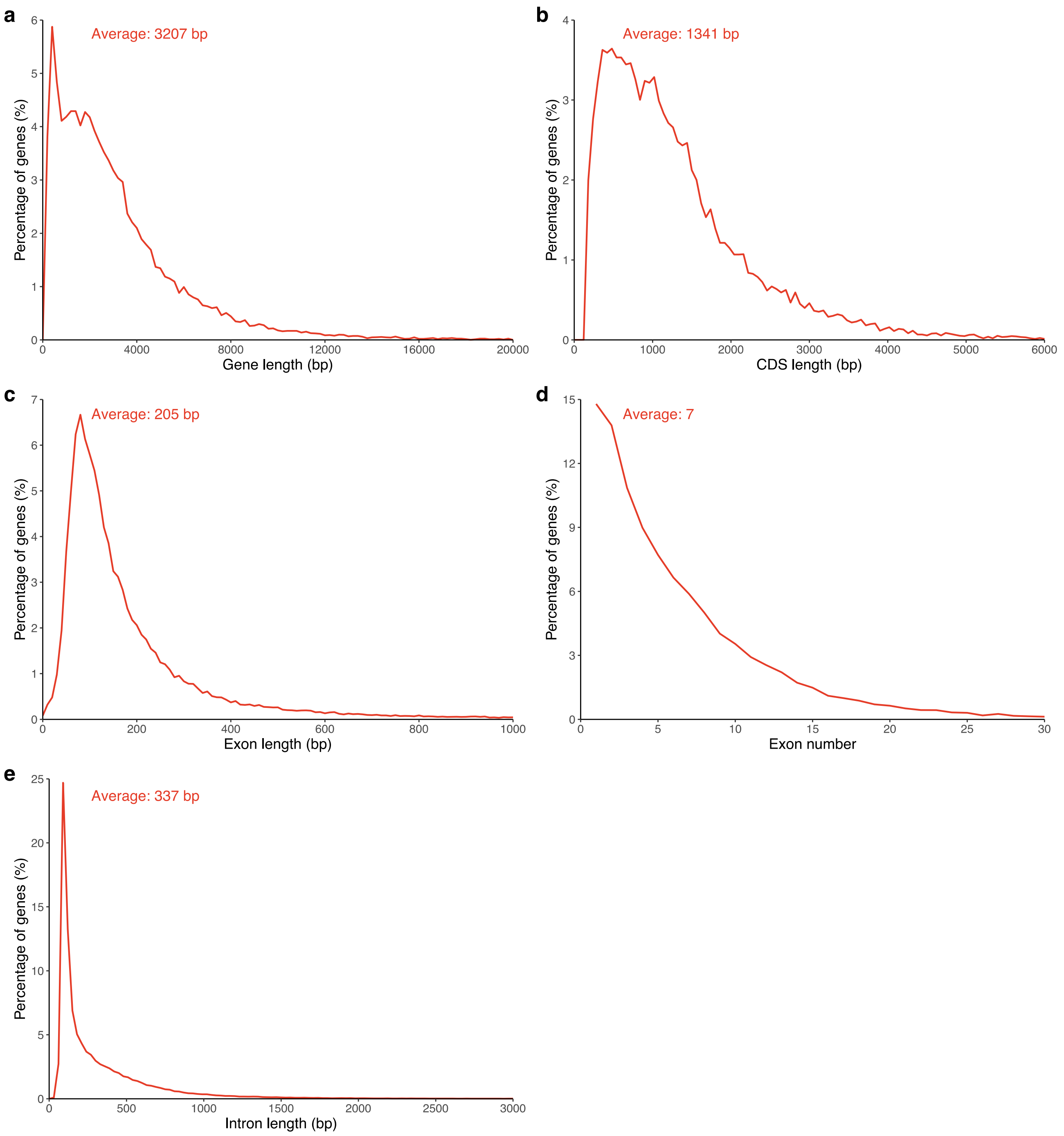

Supplementary Figure 5. Summary of predicted protein-coding genes' features in *Sonneratia alba*. (a) Gene length, (b) CDS length, (c) exon length, (d) exon number, and (e) intron length information of *S. alba*. Source data are provided as a Source Data file.

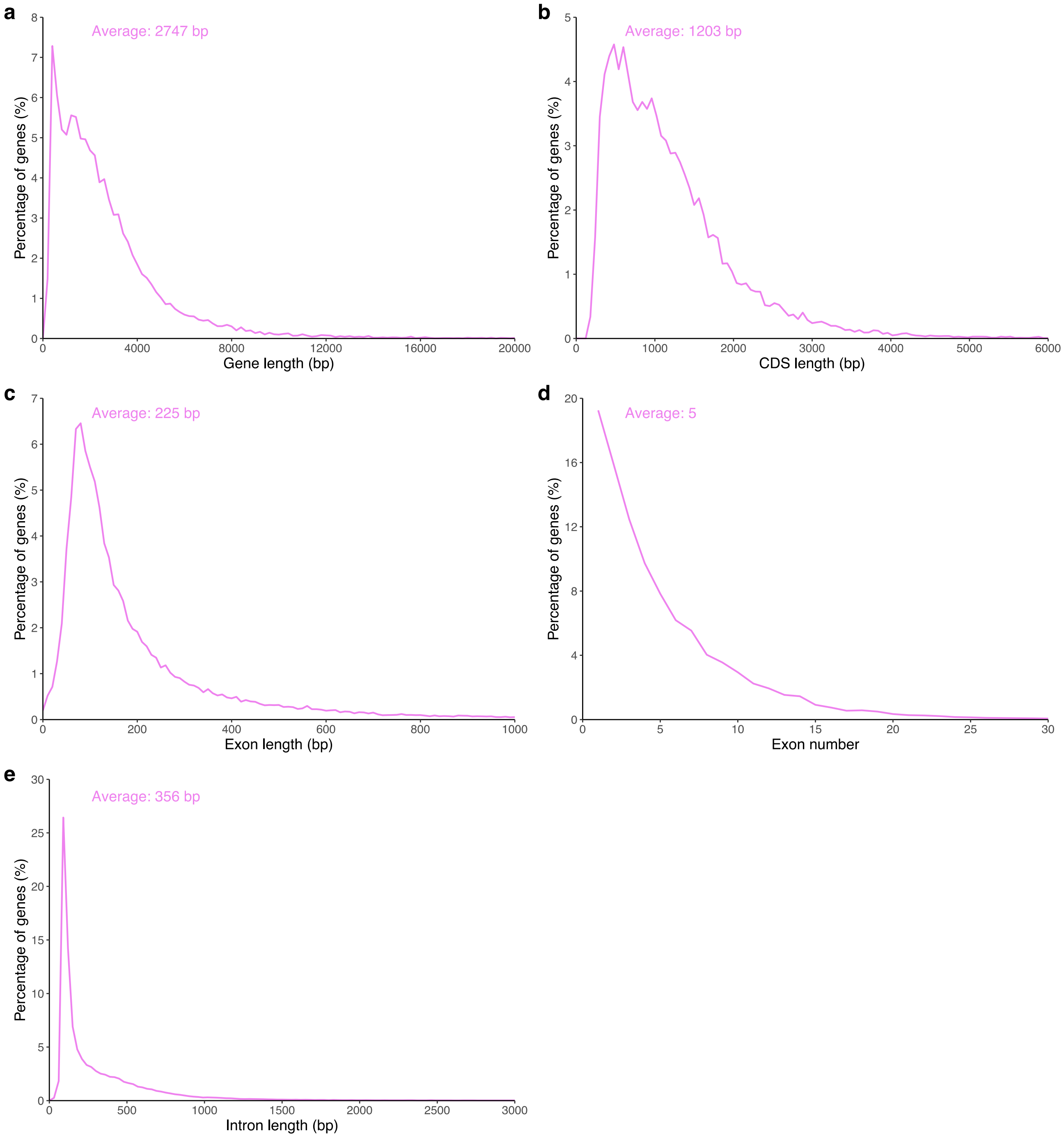

Supplementary Figure 6. Summary of predicted protein-coding genes' features in *Lagerstroemia speciosa*. (a) Gene length, (b) CDS length, (c) exon length, (d) exon number, and (e) intron length information of *L. speciosa*. Source data are provided as a Source Data file.

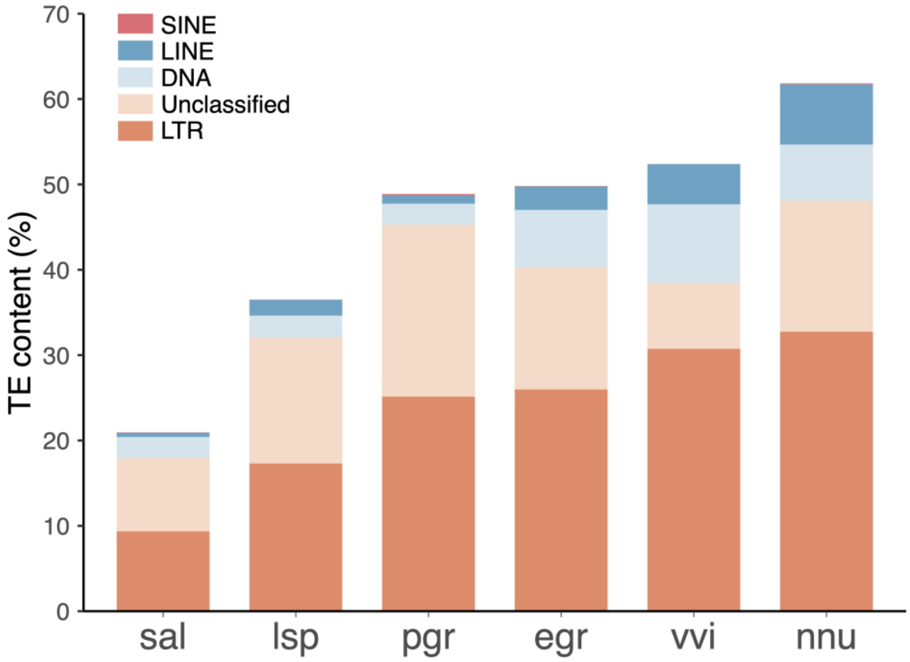

Supplementary Figure 7. Transposable element (TE) contents among *S. alba* and relatives. The proportions of different types of TEs are presented in the stacked bar plot. sal: *S. alba*; lsp: *L. speciosa*; pgr: *P. granatum*; egr: *E. grandis*; vvi: *V. vinifera*; nnu: *N. nucifera*. Source data are provided as a Source Data file.

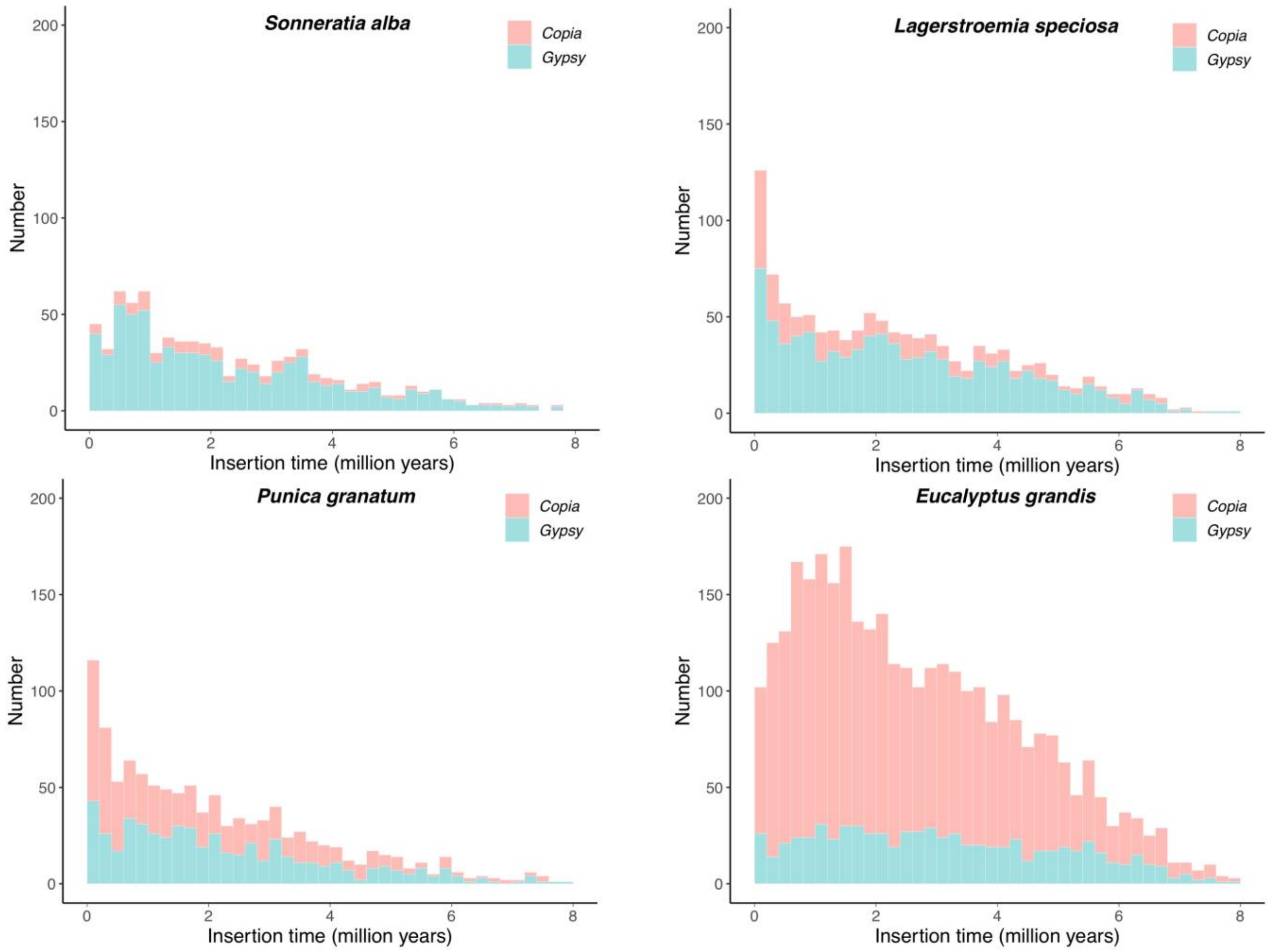

Supplementary Figure 8. Insertion time distribution of all intact LTR-RTs in *Sonneratia alba* and three relatives in Myrtales, *Lagerstroemia speciosa*, *Punica granatum*, and *Eucalyptus grandis*. The *Copia* and *Gypsy* are presented in the stacked bar with different colors. Source data are provided as a Source Data file.

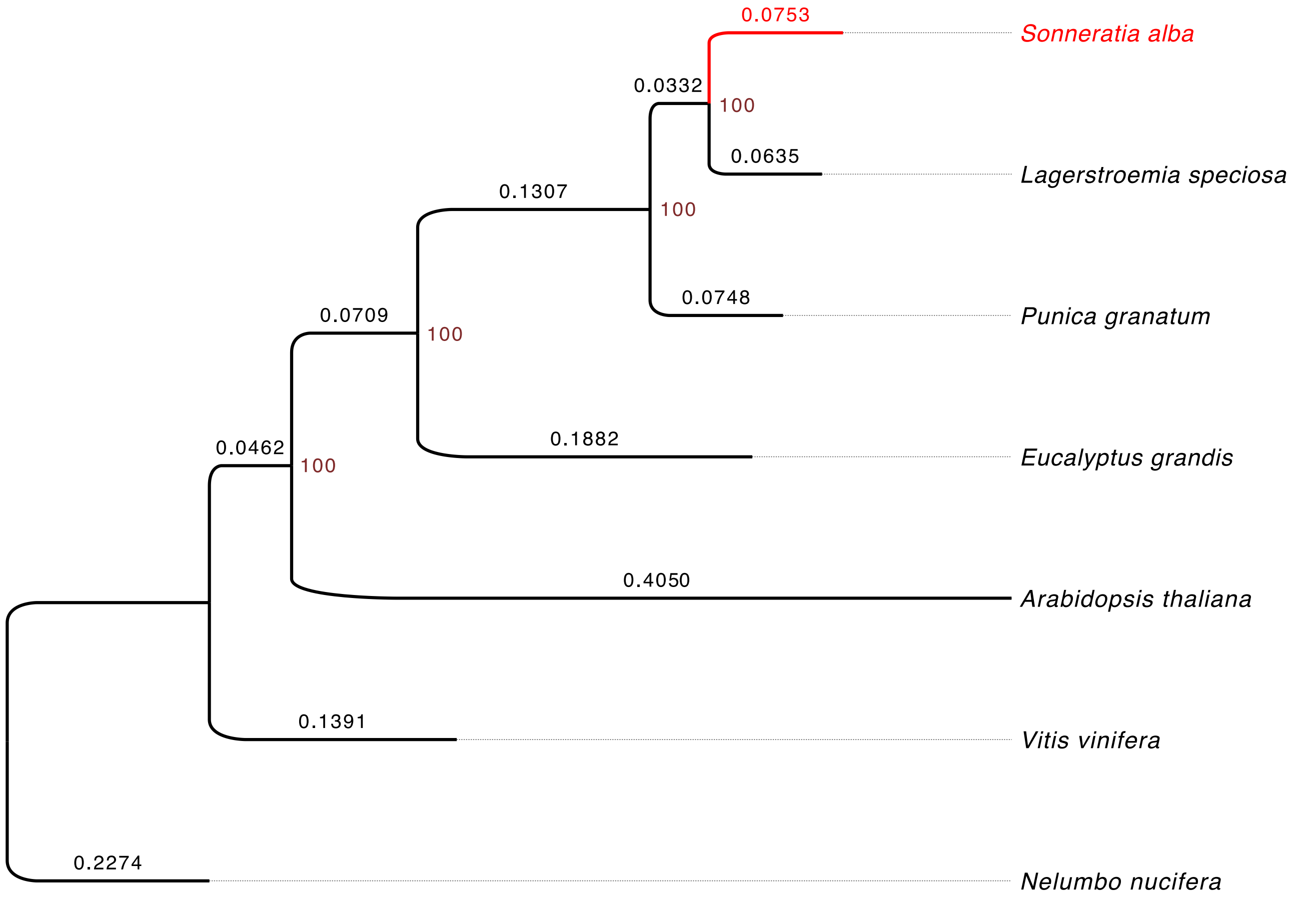

Supplementary Figure 9. Maximum likelihood tree of seven eudicot species, including *S. alba* and relatives. Black and red numbers represent branch length, and brown numbers represent bootstrap support value. The 1000 bootstraps were used, and all nodes have 100% support.

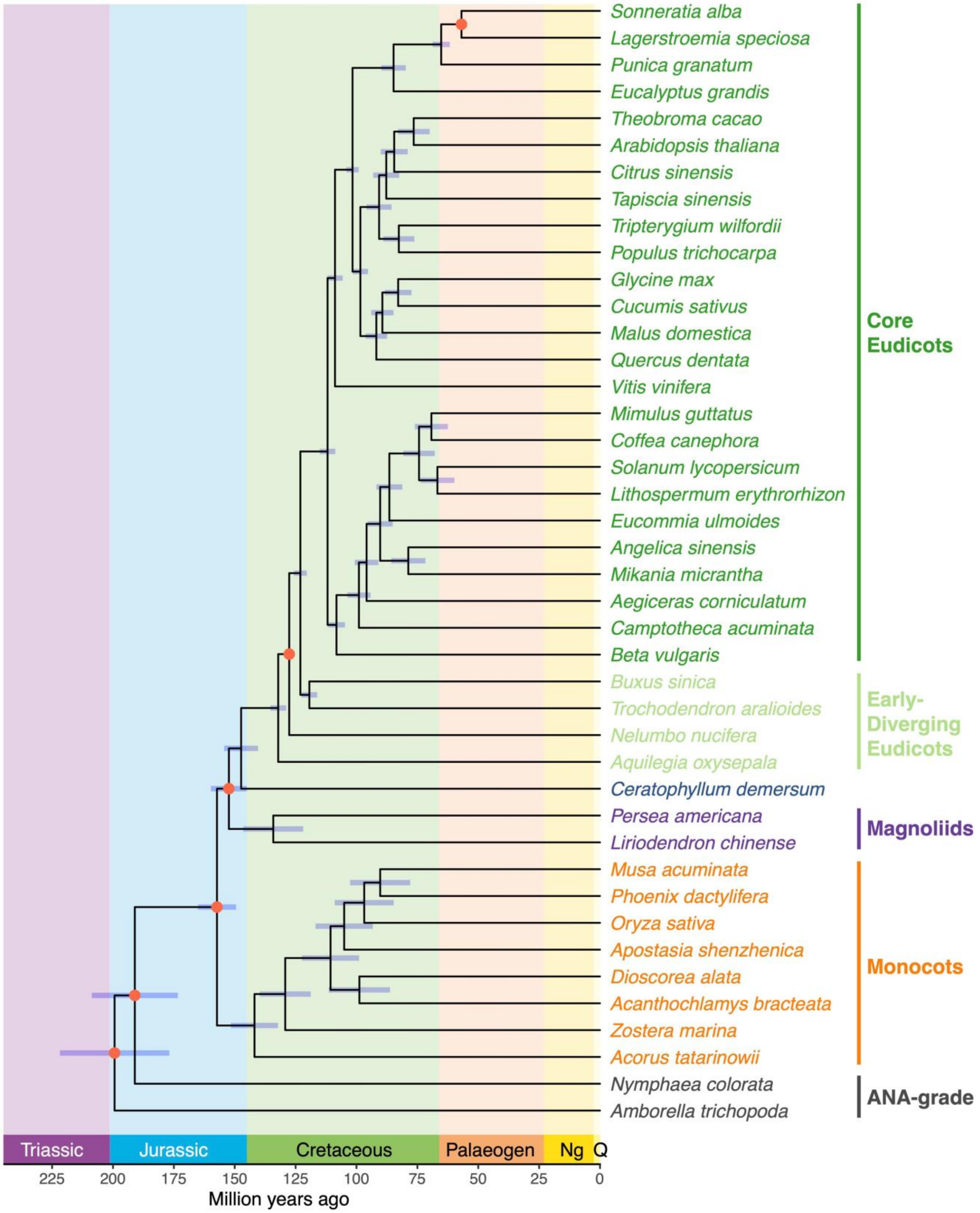

Supplementary Figure 10. Genome-scale of phylogenetic tree. The phylogenetic tree encompasses 42 angiosperms from 39 orders. The node bars represent 95% confidence intervals, with red nodes indicating fossil calibration nodes.

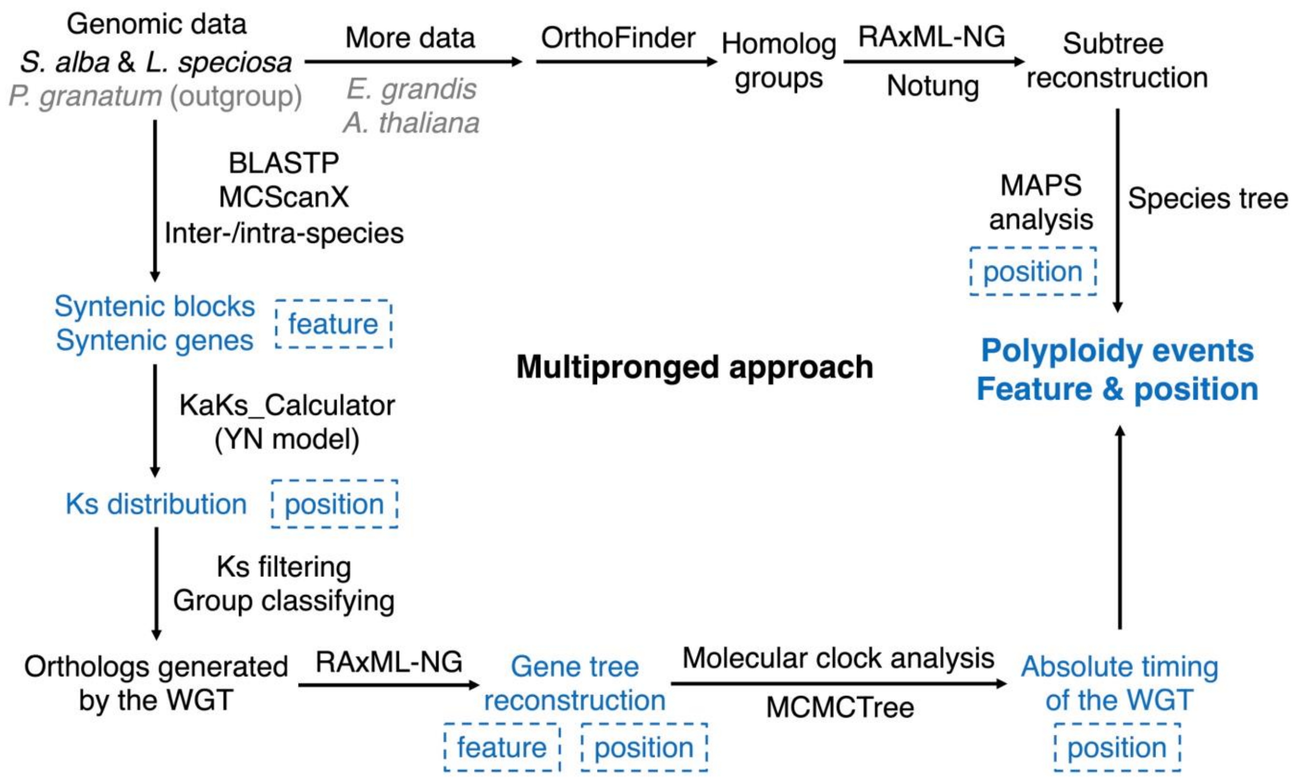

Supplementary Figure 11. Overview of workflow for the multipronged approach integrating synteny, Ks-base, and phylogenetic methodologies.

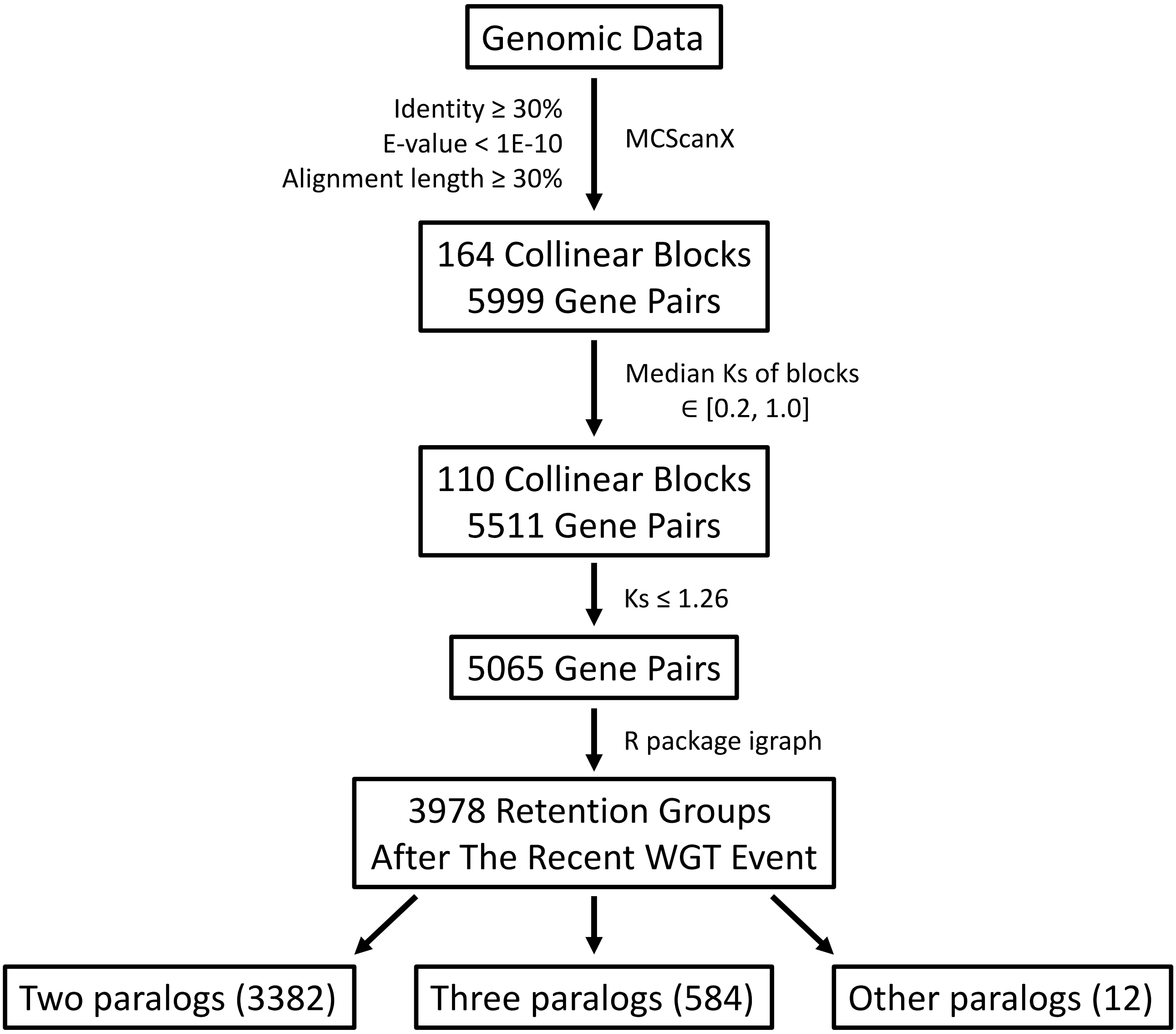

Supplementary Figure 12. Overview of workflow for the classification of the WGT retentions in *S. alba*.

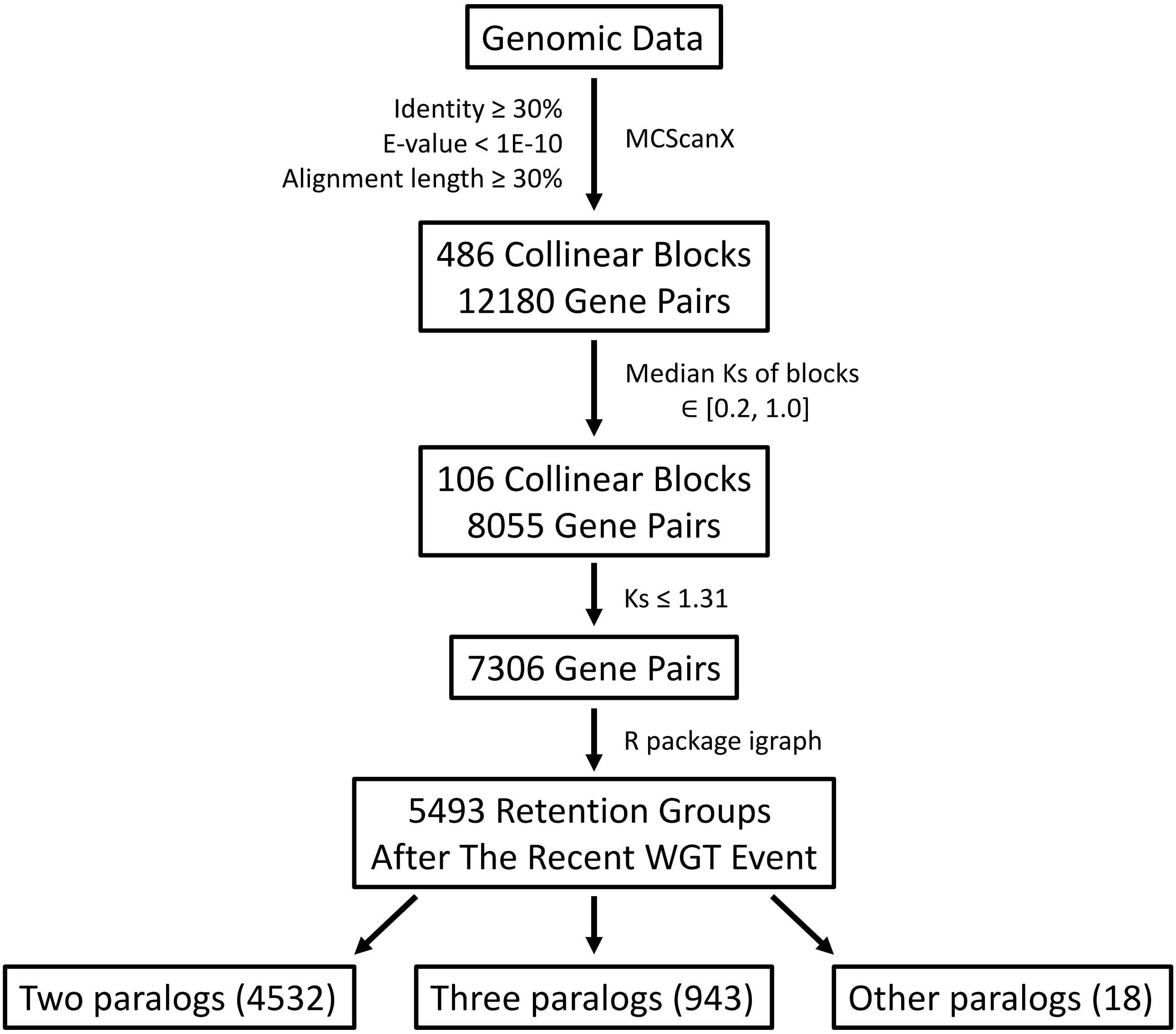

Supplementary Figure 13. Overview of workflow for the classification of the WGT retentions in *L. speciosa*.

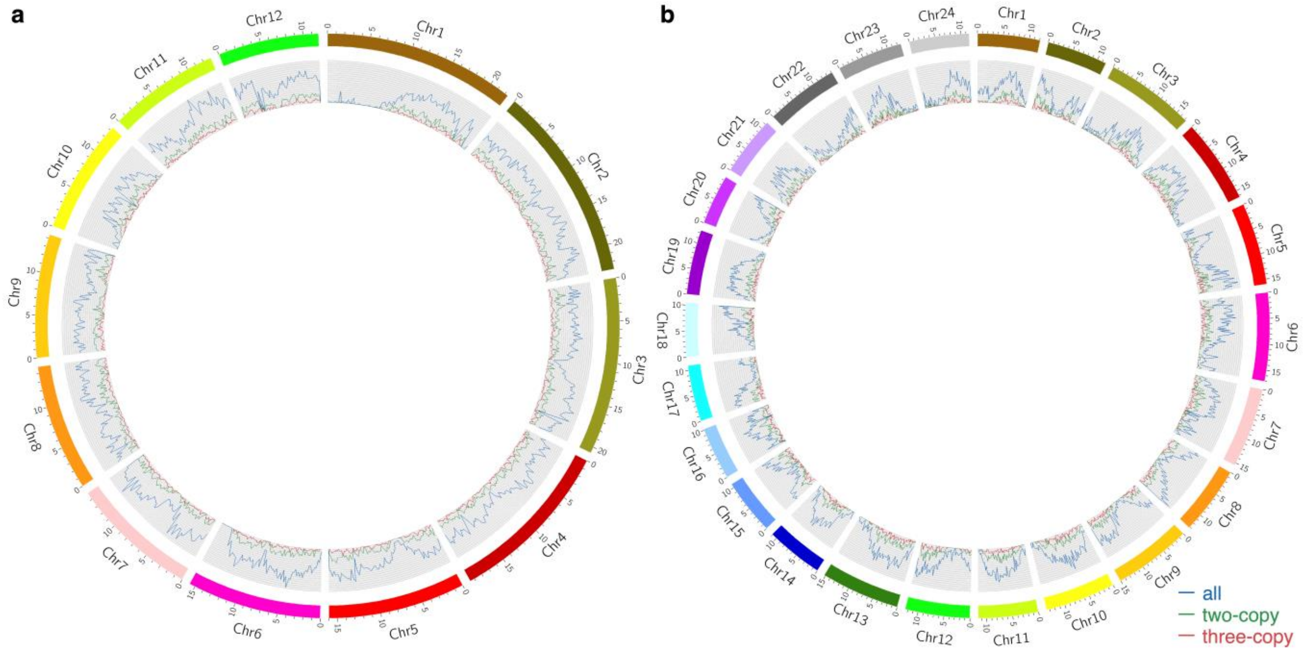

Supplementary Figure 14. Distribution of WGT retention gene densities. (a) *S. alba*; (b) *L. speciosa*. The blue lines represent the densities of all genes, the green lines represent the densities of genes belonging to two-copy duplicated gene groups, and red lines represent the densities of genes belonging to three-copy duplicated gene groups. Gene density ranges from 0 to 50 per 200 Kb. Source data are provided as a Source Data file.

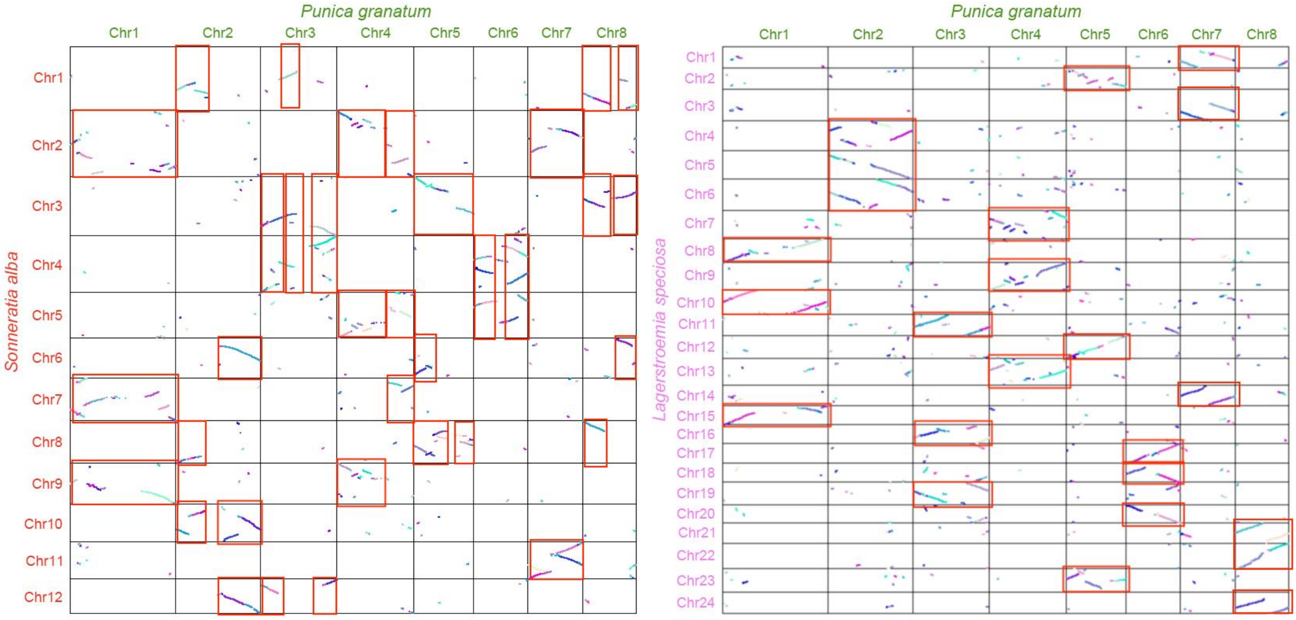

Supplementary Figure 15. Illustration of syntenic orthologues. It reflects the overall synteny relationship with a 3:1 ratio between *S. alba vs. P. granatum* (left panel), *L. speciosa vs. P. granatum* (right panel), respectively.

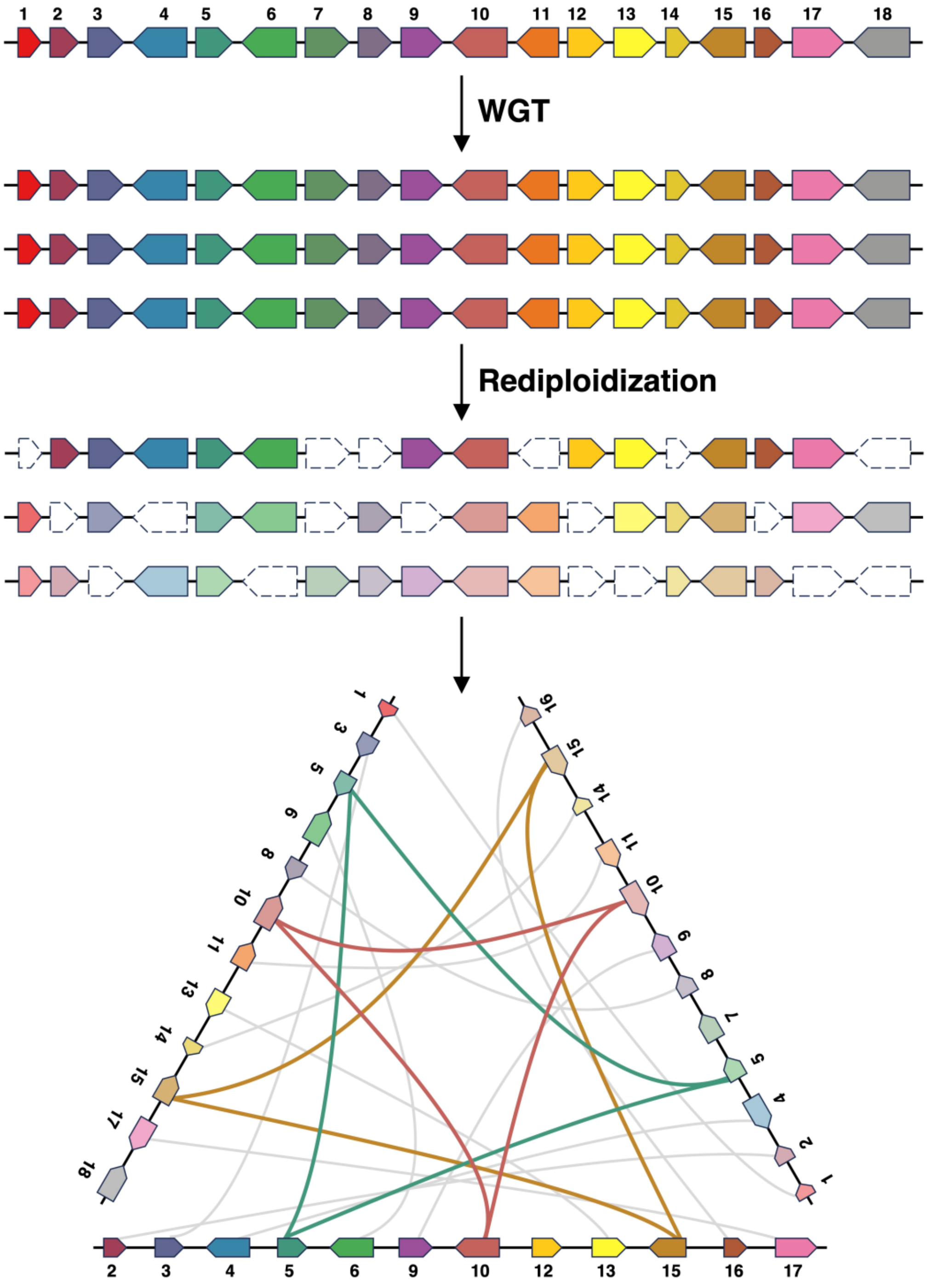

Supplementary Figure 16. The pattern of collinear gene changes during whole-genome triplication (WGT) and the subsequent rediploidization process. This explains why the WGT event can be inferred from specific existing collinear genes, even in the presence of the rediploidization process.

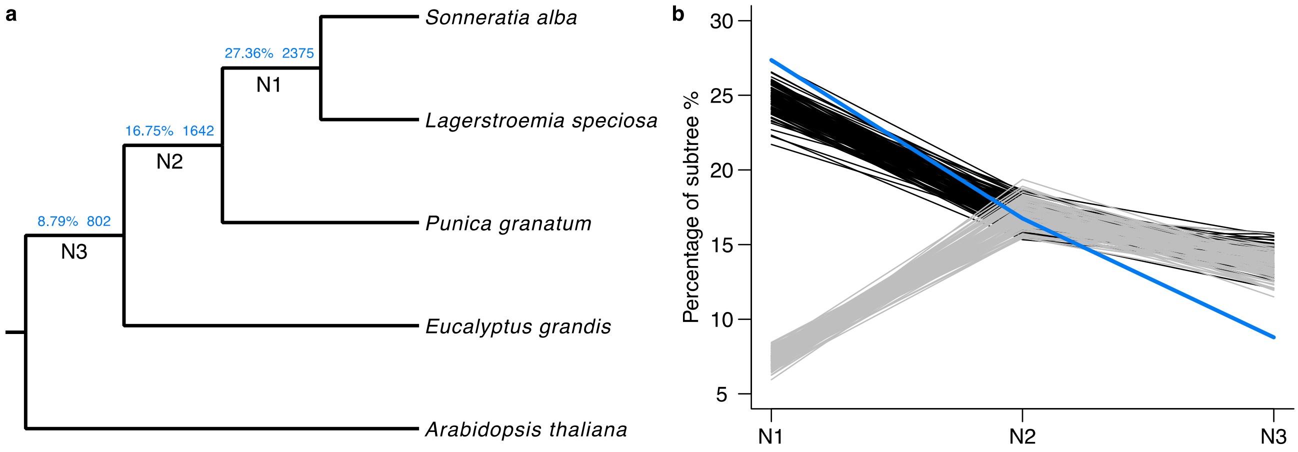

Supplementary Figure 17. MAPS results on the associated phylogeny. (a) Percentages and numbers of subtrees with a gene duplication shared by all species descended from each node are shown on the phylogenetic tree. (b) Percentages of subtrees contain a gene duplication shared by all species descended from each node. The results are portrayed for observed data (a blue line), 100 replicates of null simulations (gray lines), and positive simulations (black lines). Source data are provided as a Source Data file.

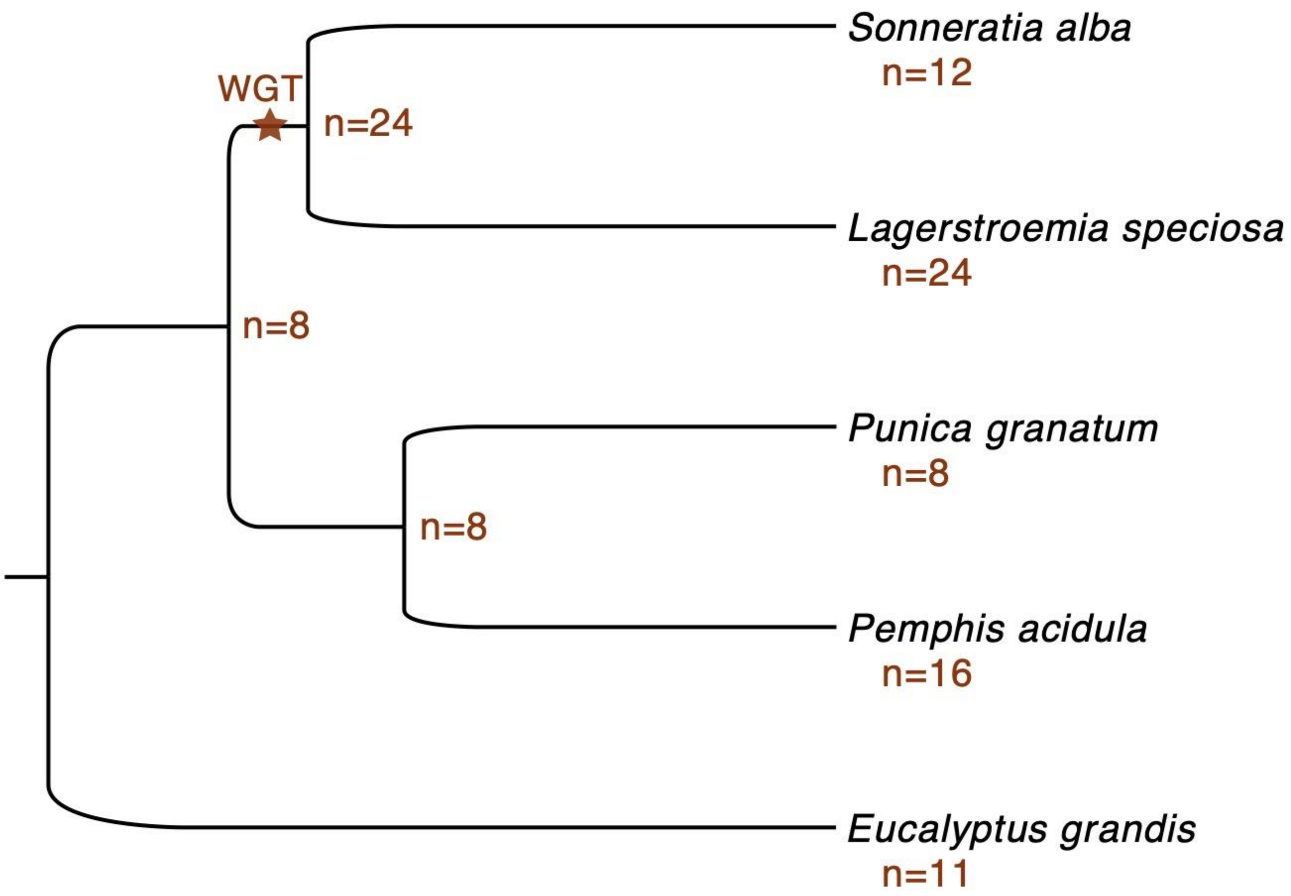

Supplementary Figure 18. Maximum likelihood estimates of ancestral chromosome numbers. The brown numbers indicate haploid chromosome numbers (*n*) of nodes. The star represents a whole-genome triplication event.

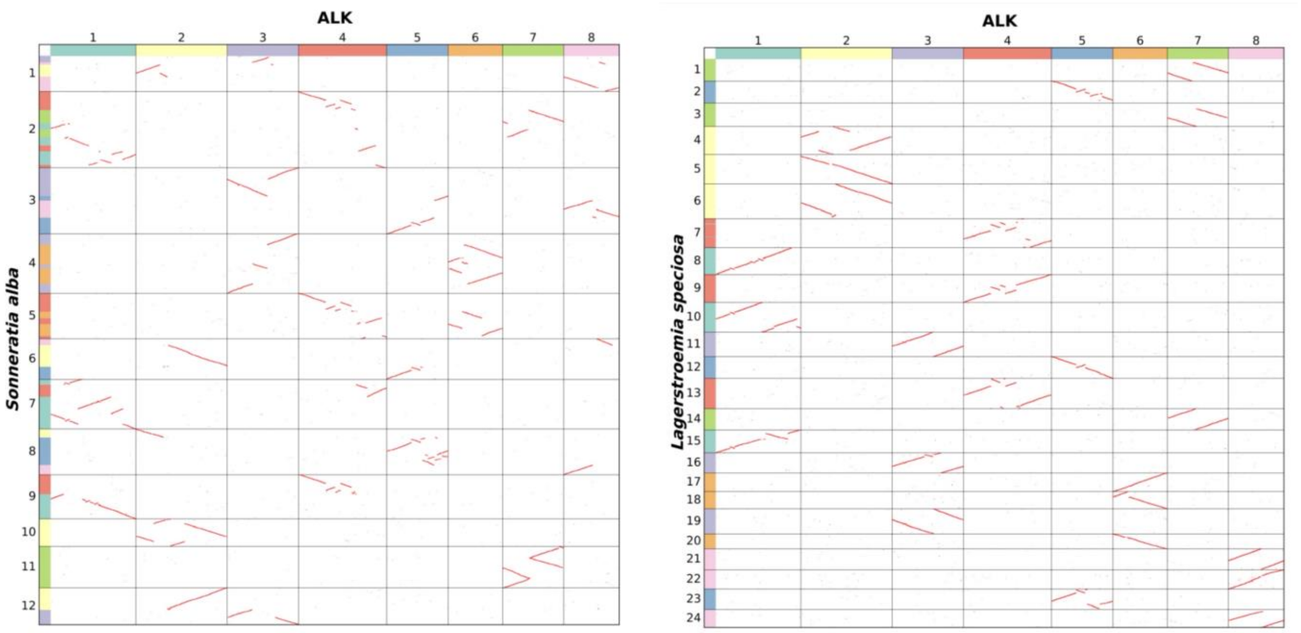

Supplementary Figure 19. Dotplots of homologous blocks between *S. alba* and ancestral Lythraceae karyotype (ALK), and *L. speciosa* and ALK. The ALK was used as the reference genome.

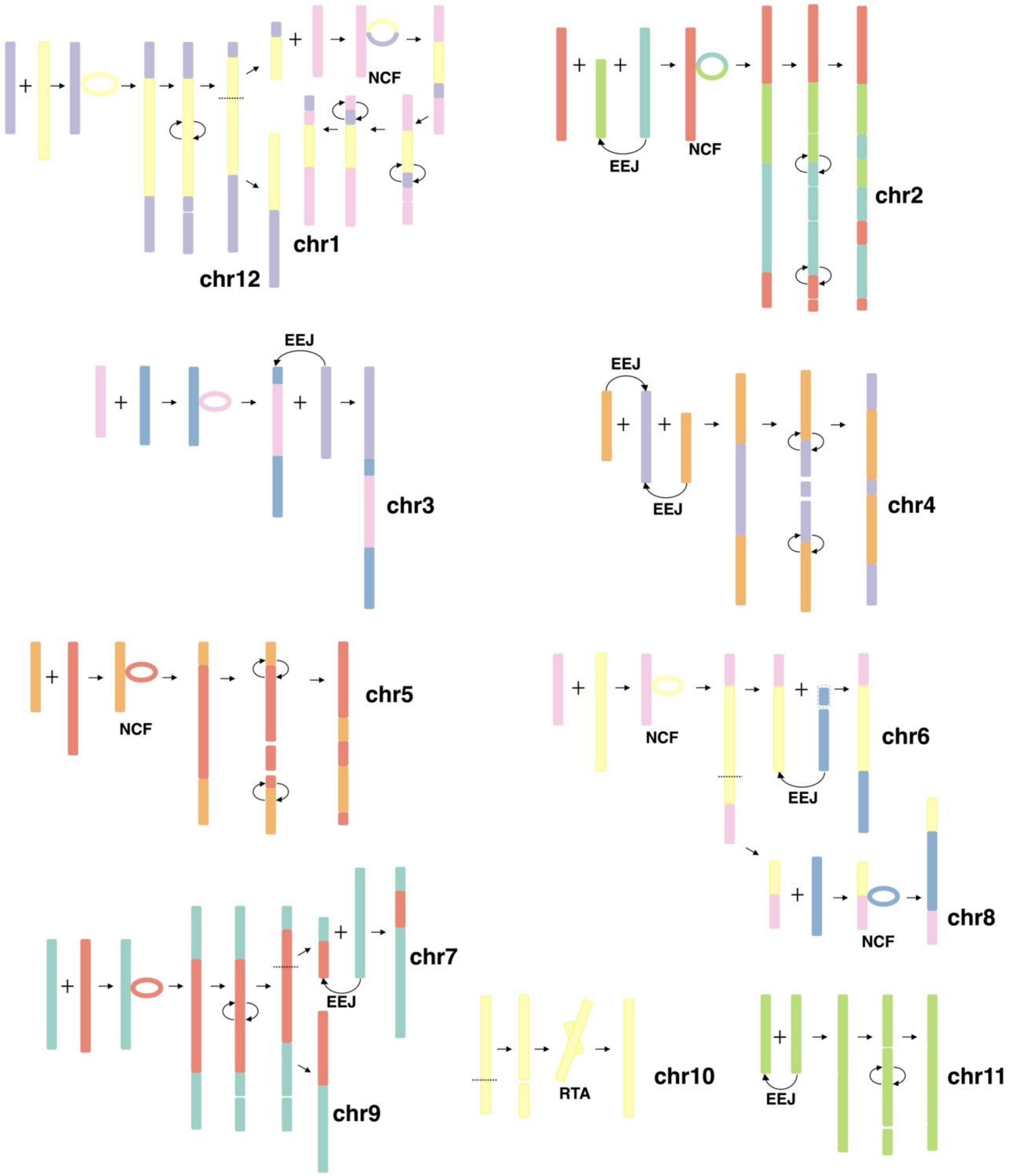

Supplementary Figure 20. Karyotype evolution of *S. alba*. The evolutionary history of each chromosome was inferred using WGDI. RTA, EEJ and NCF are the abbreviations for reciprocally translocated chromosome arms, end-end joining, and nested chromosome fusion. The dashed line indicates chromosome fission, and the exchange icon indicates chromosome inversion. The chromosome inversions within the same chromosome of the ALK are not depicted in the schematic diagram, except for chromosome 11. The colors, consistent with the ALK in Fig. 2e, represent different ancestral chromosomes.

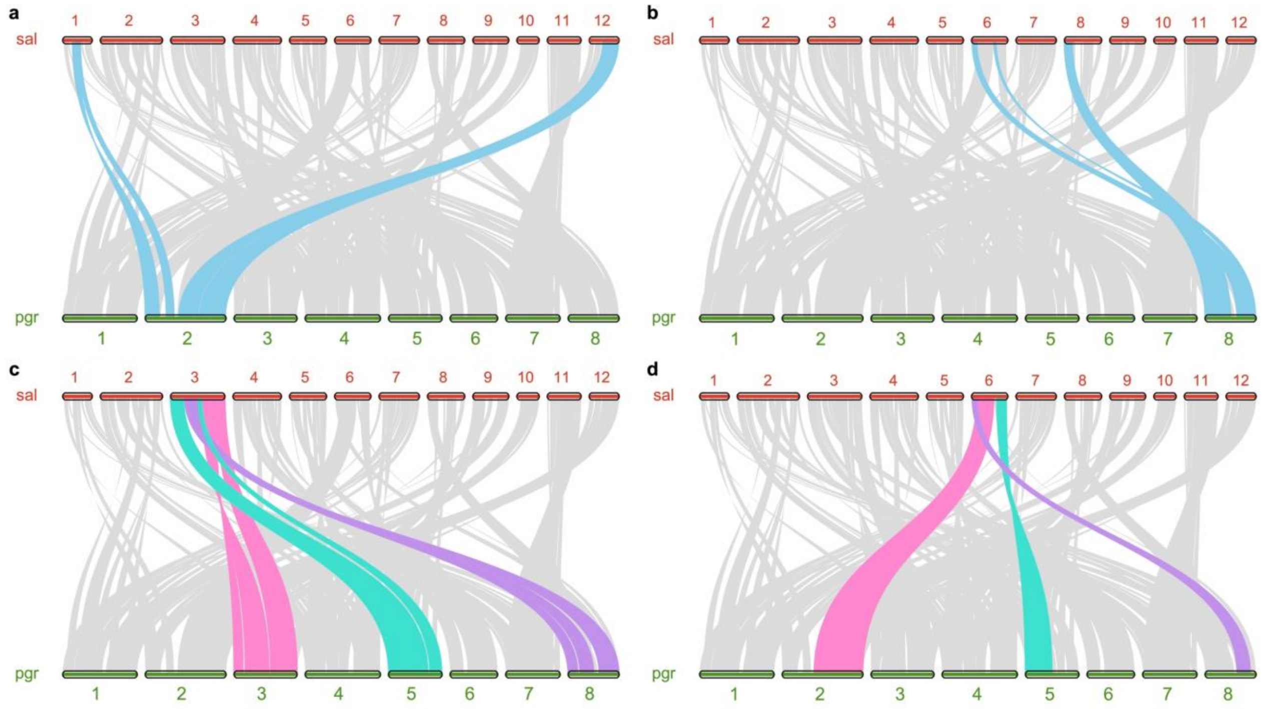

Supplementary Figure 21. Macrosynteny patterns between *S. alba* and *P. granatum*. The syntenic regions are highlighted in gray. Various colors represent distinct fission and fusion events. In *S. alba*, Chromosomes 1 and 12 resulted from fission (a); Chromosomes 6 and 8 originated from fission (b); Chromosome 3 resulted from fusion (c); Chromosome 6 resulted from fusion (d). sal: *S. alba*, pgr: *P. granatum*. Source data are provided as a Source Data file.

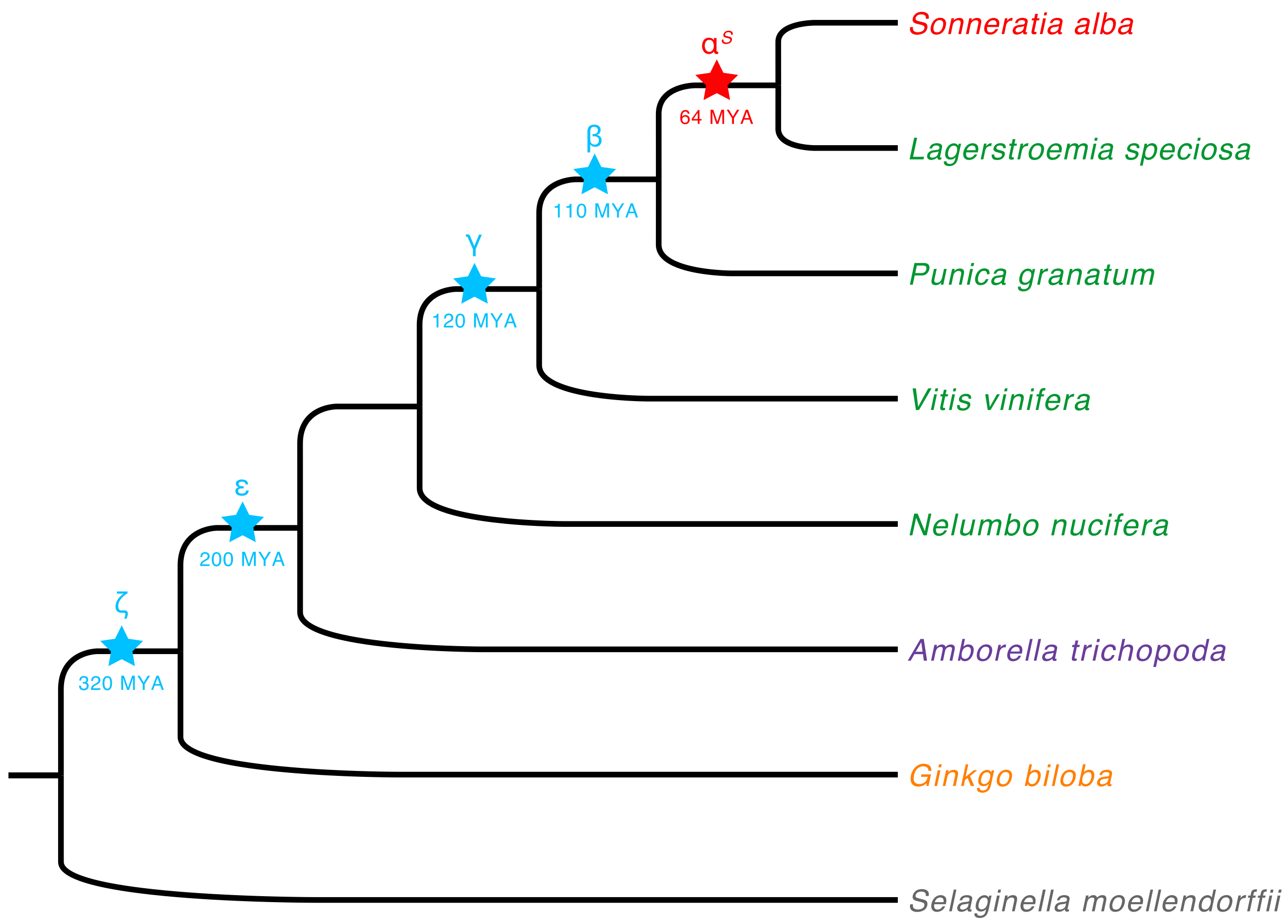

Supplementary Figure 22. Polyploidy events in the evolutionary history from ancestral seed plants to *Sonneratia*. The tree shows the phylogenetic relationship. Polyploidy events are superimposed on the tree, including ζ occurring in the common ancestor of extant seed plants, ε in the common ancestor of extant angiosperms, γ in the common ancestor of core eudicots, β in the common ancestor of Lythraceae, α^S in the common ancestor of *Sonneratia* and *Lagerstroemia*.

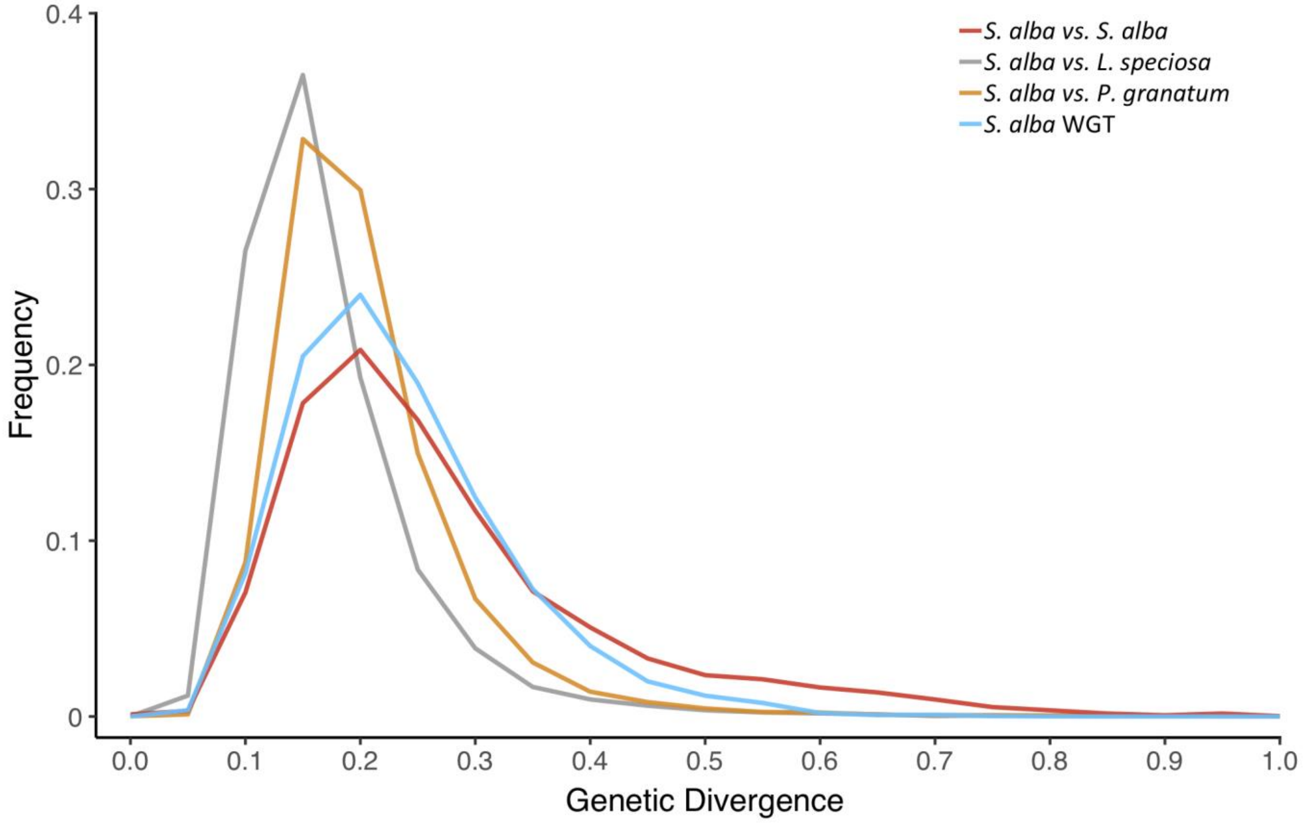

Supplementary Figure 23. Genetic divergence of paralogous genes within the same species and orthologous genes from pairs of species. The genetic divergence was estimated by the Kimura two-parameter (K2P) method. The genetic divergence of paralogous gene pairs generated by the WGT event in *S. alba* is shown by a blue line. Source data are provided as a Source Data file.

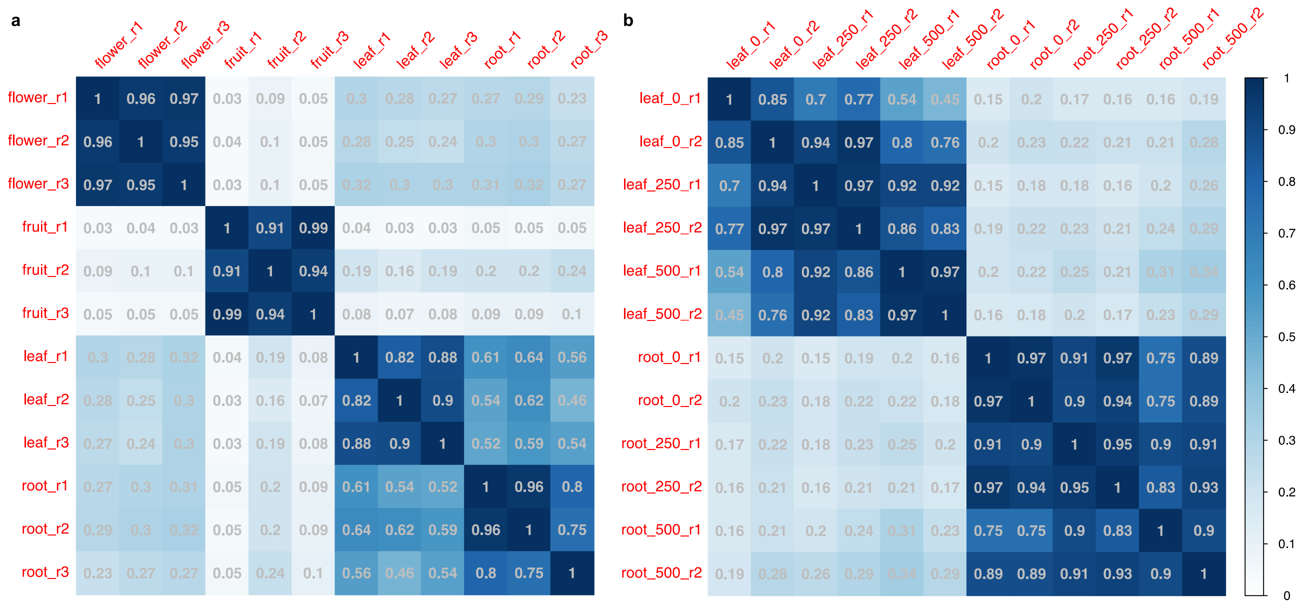

Supplementary Figure 24. Gene expression correlations in *S. alba*. (a) Expression correlations among leaf, root, flower, fruit tissues. (b) Expression correlations among leaf and root tissues under different high salinity conditions. Pearson's correlation plot visualizes the correlation coefficients by the R package corrplot. The scale bar on the right represents the range of the value displayed. Source data are provided as a Source Data file.

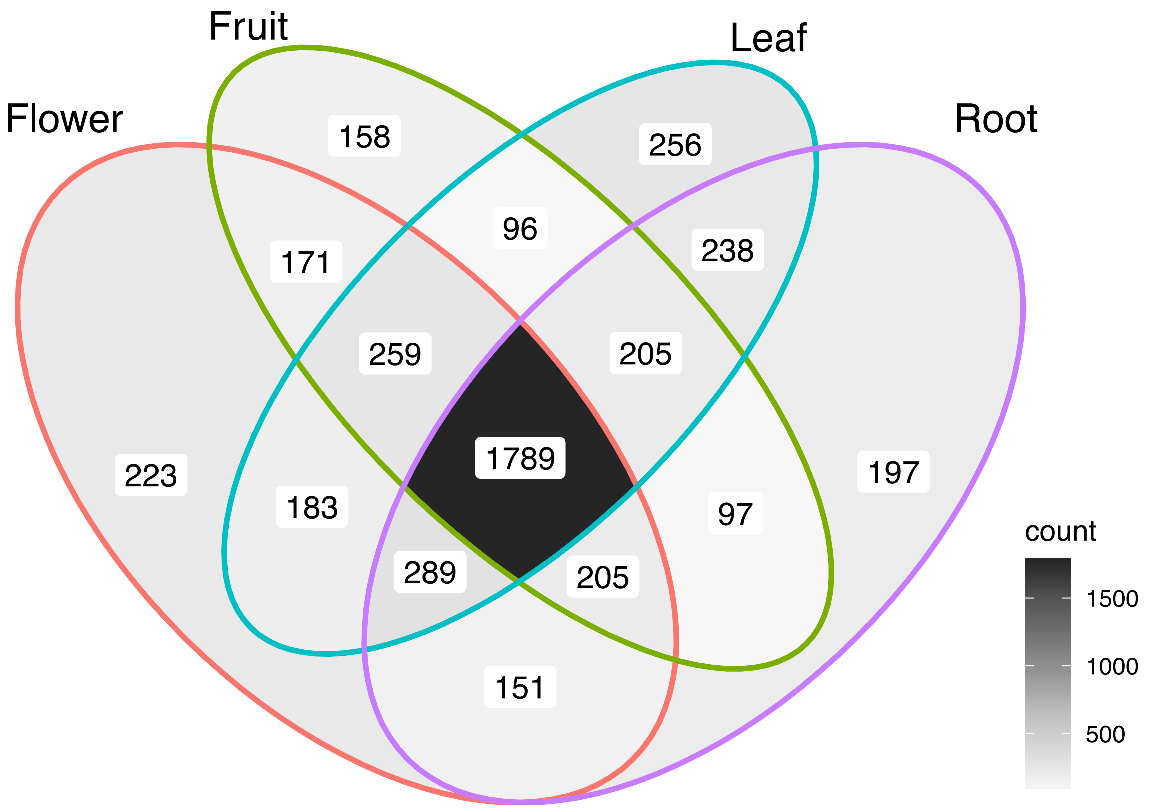

Supplementary Figure 25. The number of differentially expressed gene pairs (DEGPs) among leaf, root, flower, and fruit tissues in *S. alba*. Source data are provided as a Source Data file.

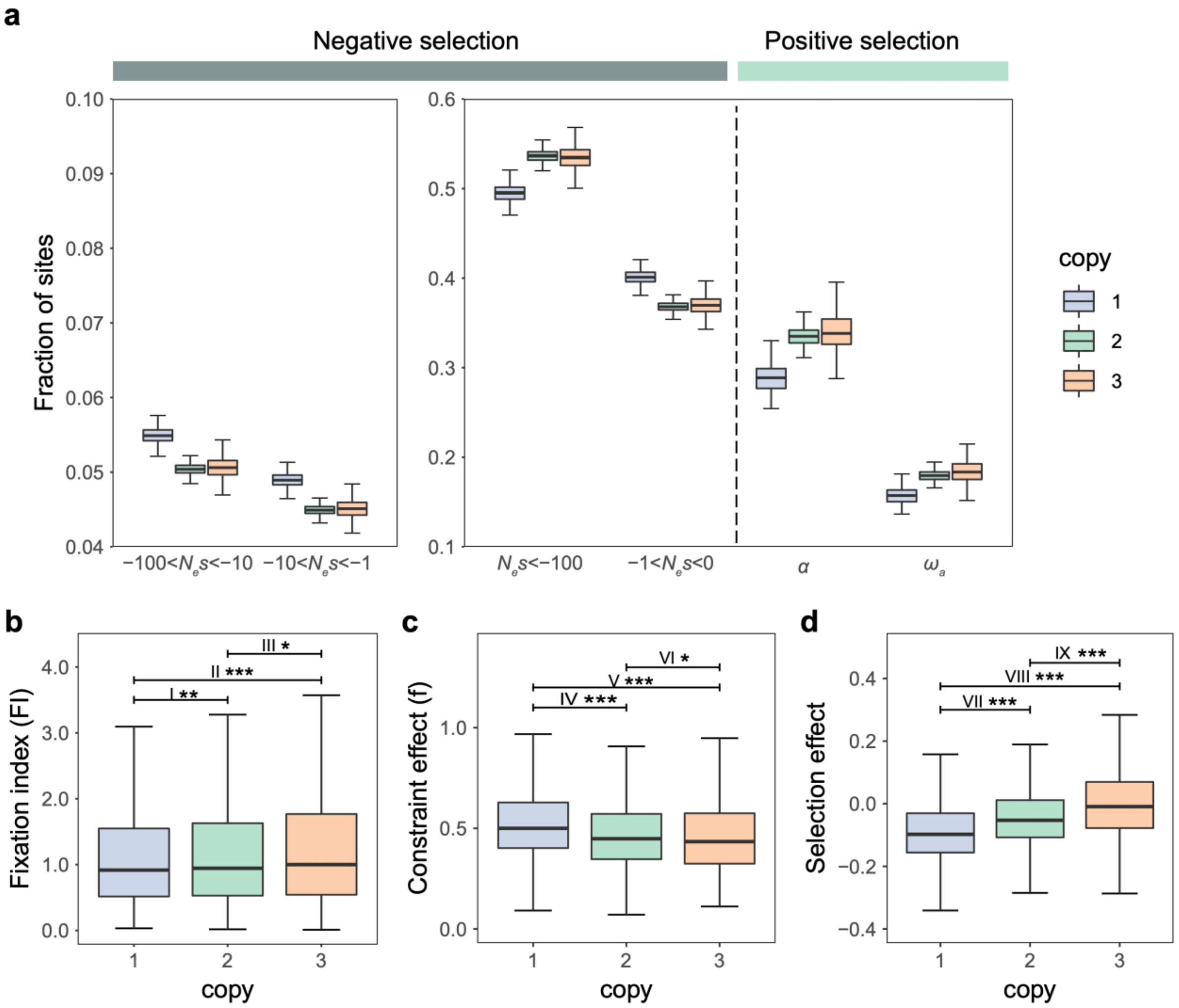

Supplementary Figure 26. Natural selection patterns among different-copy retention groups. (a) The inferred distribution of fitness effects (N_es), proportion of adaptive divergence (α), and rate of adaptive substitution relative to neutral divergence (ω_a) for different-copy retention groups generated by the WGT event. Each distribution was estimated based on 200 bootstrap replicates. Traditional MK test with fixation index (b), constraint effect (c), and selection effect (d) for these different-copy retention groups. The two-tailed t-test was applied to test for all pairwise differences. *P*-values are indicated by single asterisks (*P*-value < 0.05), double asterisks (*P*-value < 0.01) or triple asterisks (*P*-value < 0.001). I** (*P*-value = 9.3×10^{-3}), II*** (*P*-value = 5.2×10^{-4}), III* (*P*-value = 4.6×10^{-2}), VI* (*P*-value = 1.5×10^{-2}), IV ***, V***, VII***, VIII***, and IX*** (*P*-value < 1×10^{-15}) represent the significantly different values for pairwise comparisons between groups. Box edges indicate upper and lower quartiles, centerlines indicate median values, and whiskers extend to 1.5 times the interquartile range. The number of genes in each retention group (one-copy, two-copy, and three-copy) was 3,439, 4,832, and 1,171, respectively. Samples of this population were collected from Davao, Philippines, while Cebu, Philippines in Fig. 4. Source data are provided as a Source Data file.

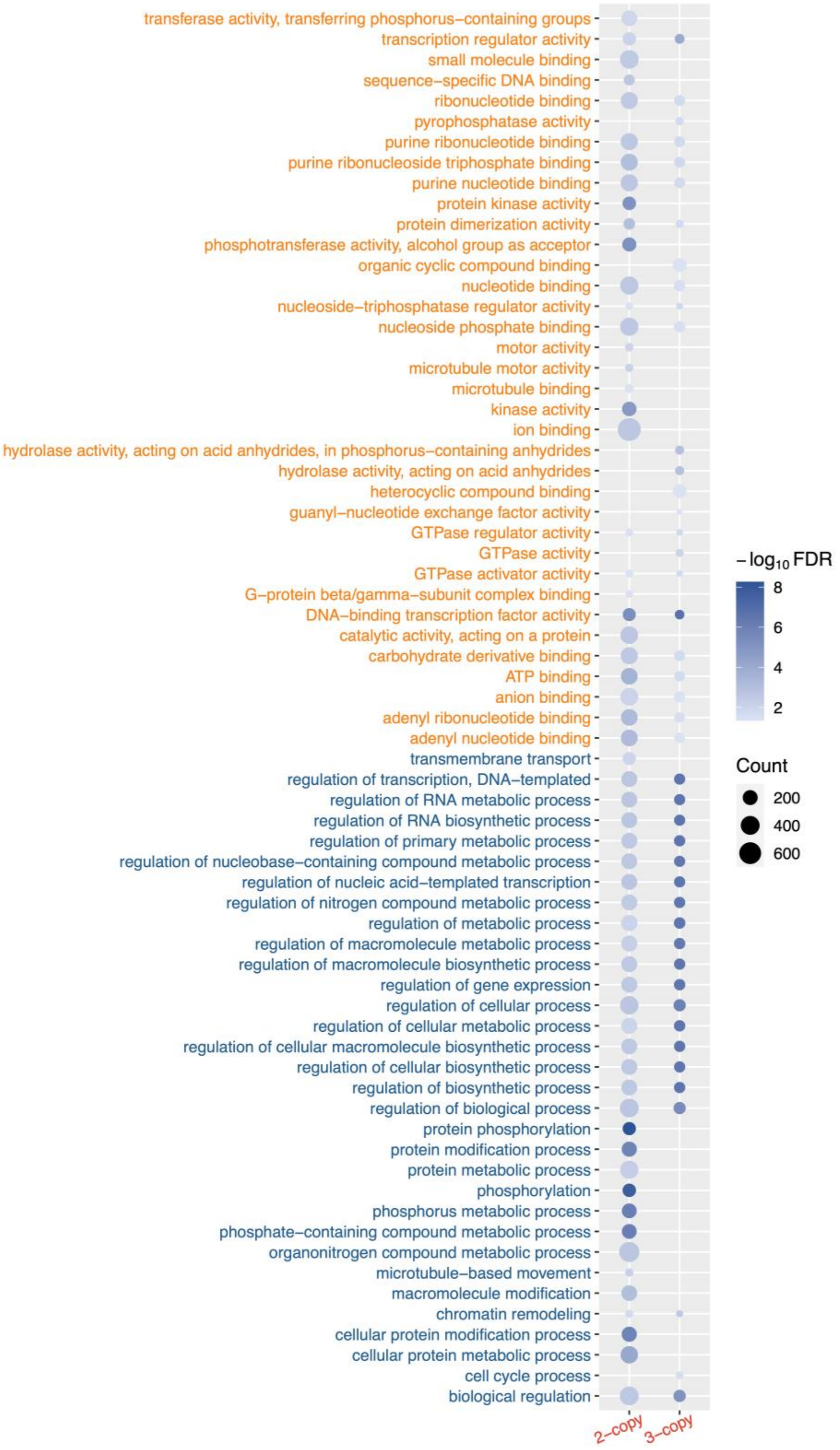

Supplementary Figure 27. Gene Ontology enrichment among the WGT retained gene duplicates compared with single genes in *S. alba*. The size and color of the bubbles represent the number of retention groups and FDR value. A darker bubble color on the scale indicates a smaller FDR value. The retention groups are shown on the *x*-axis and GO categories are shown on the *y*-axis. The orange and blue labels indicate molecular function and biological process, respectively. Source data are provided as a Source Data file.

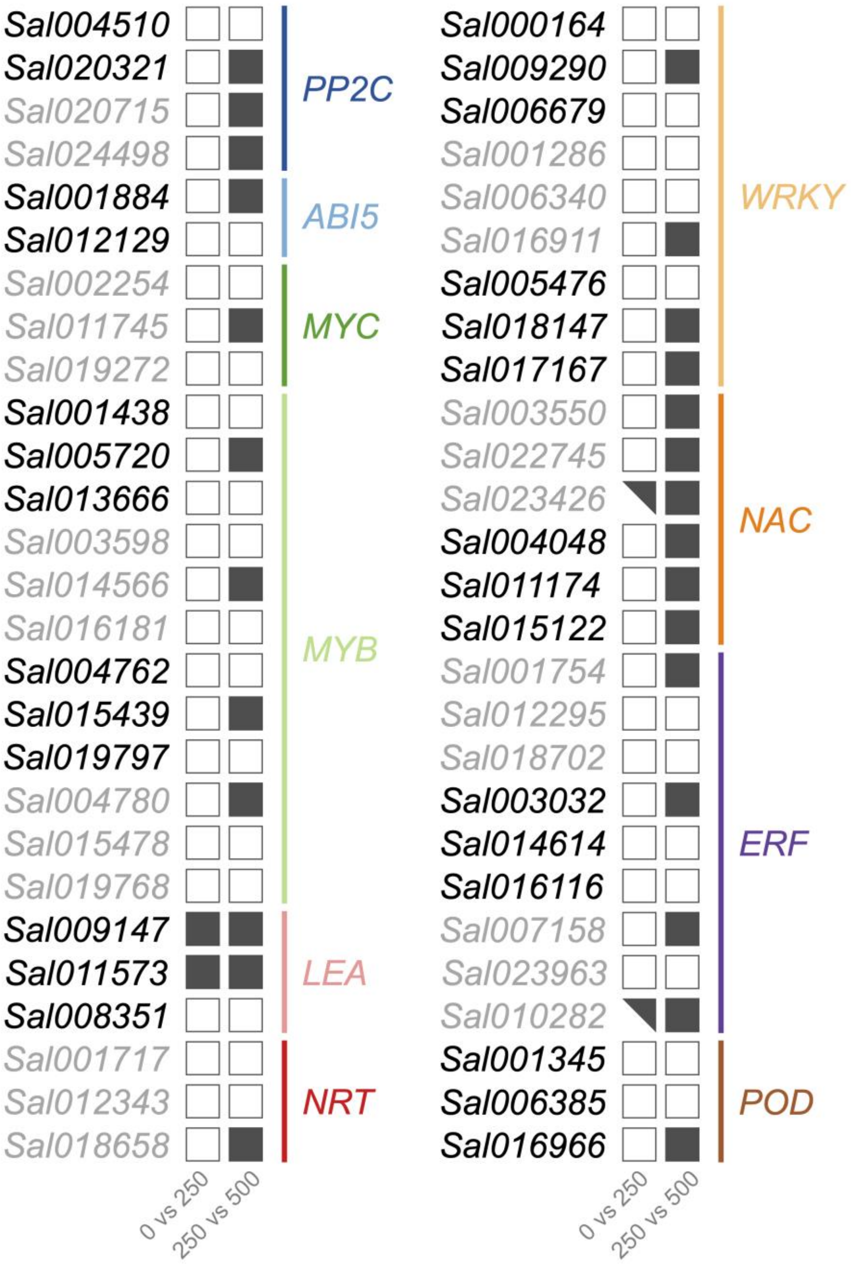

Supplementary Figure 28. The expression pattern of WGT retentions in leaf tissue across salinity conditions. Solid squares represent up-regulation, solid triangles represent down-regulation, hollow squares represent no significant difference. Continuous genes with the same grayscale color are from the same retention groups generated by the WGT event.

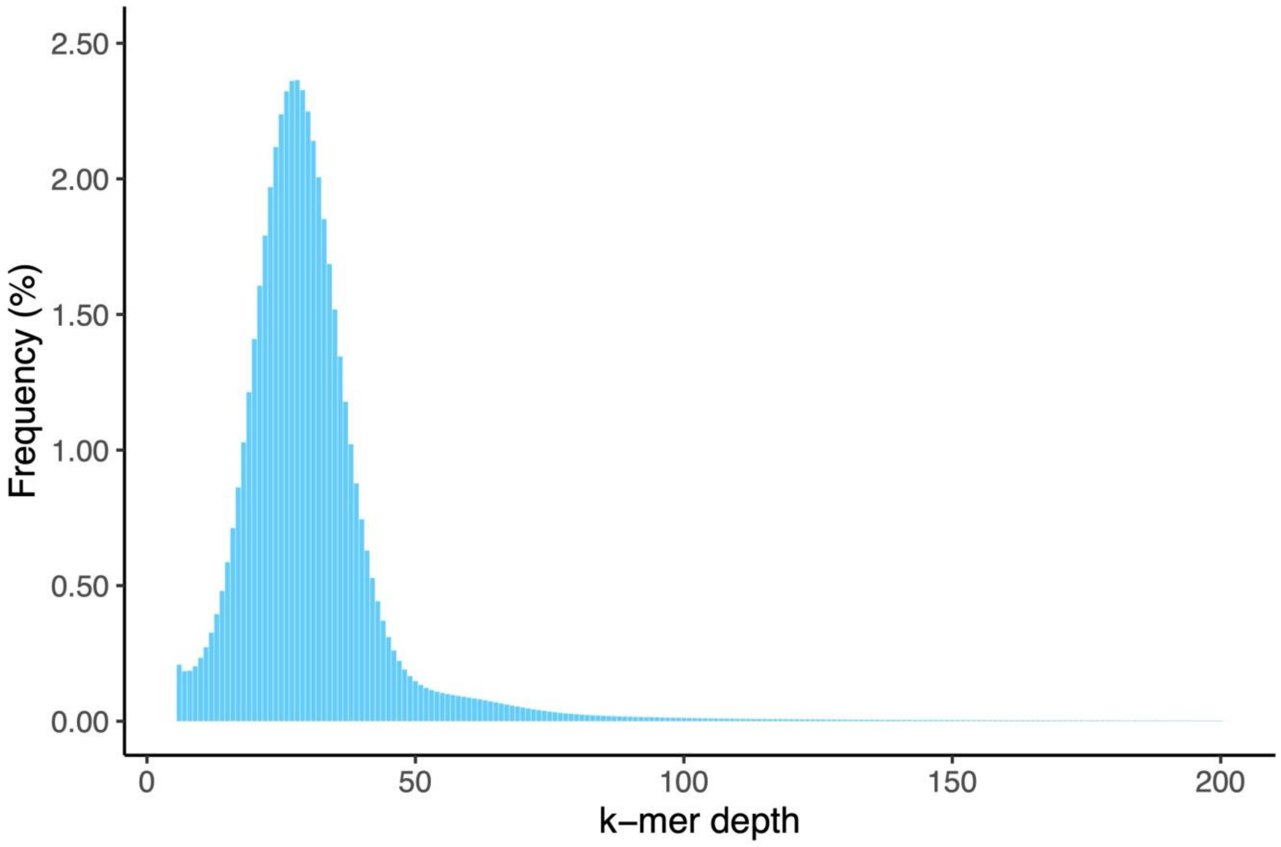

Supplementary Figure 29. The k-mer distribution of the *S. alba* genome. Source data are provided as a Source Data file.

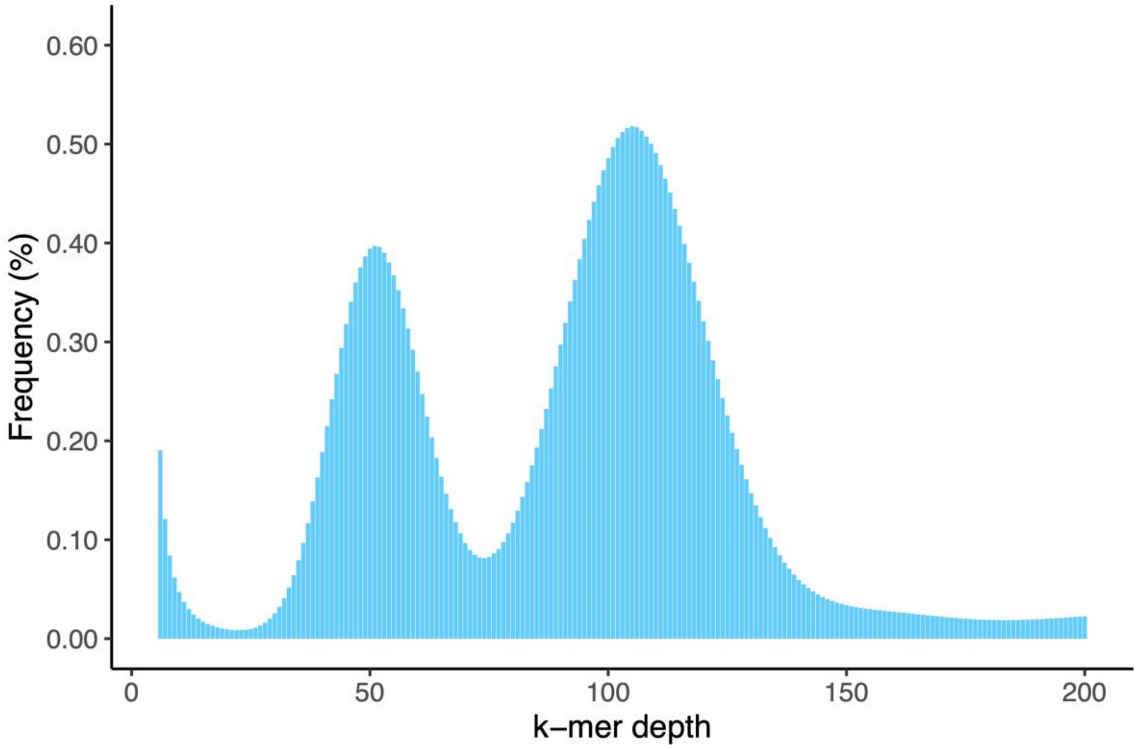

Supplementary Figure 30. The k-mer distribution of the *L. speciosa* genome. Source data are provided as a Source Data file.

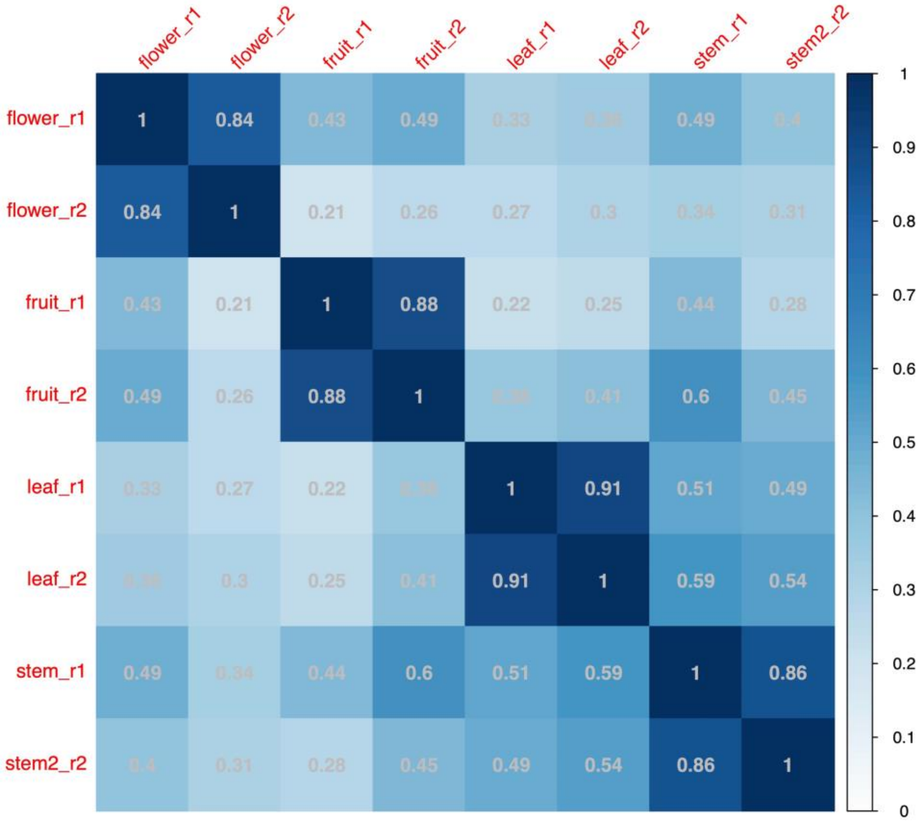

Supplementary Figure 31. Gene expression correlations in *L. speciosa*. Expression correlations among flower, fruit, leaf, stem tissues. Pearson’s correlation plot visualizes the correlation coefficients by the R package corrplot. The scale bar on the right represents the range of the value displayed. Source data are provided as a Source Data file.

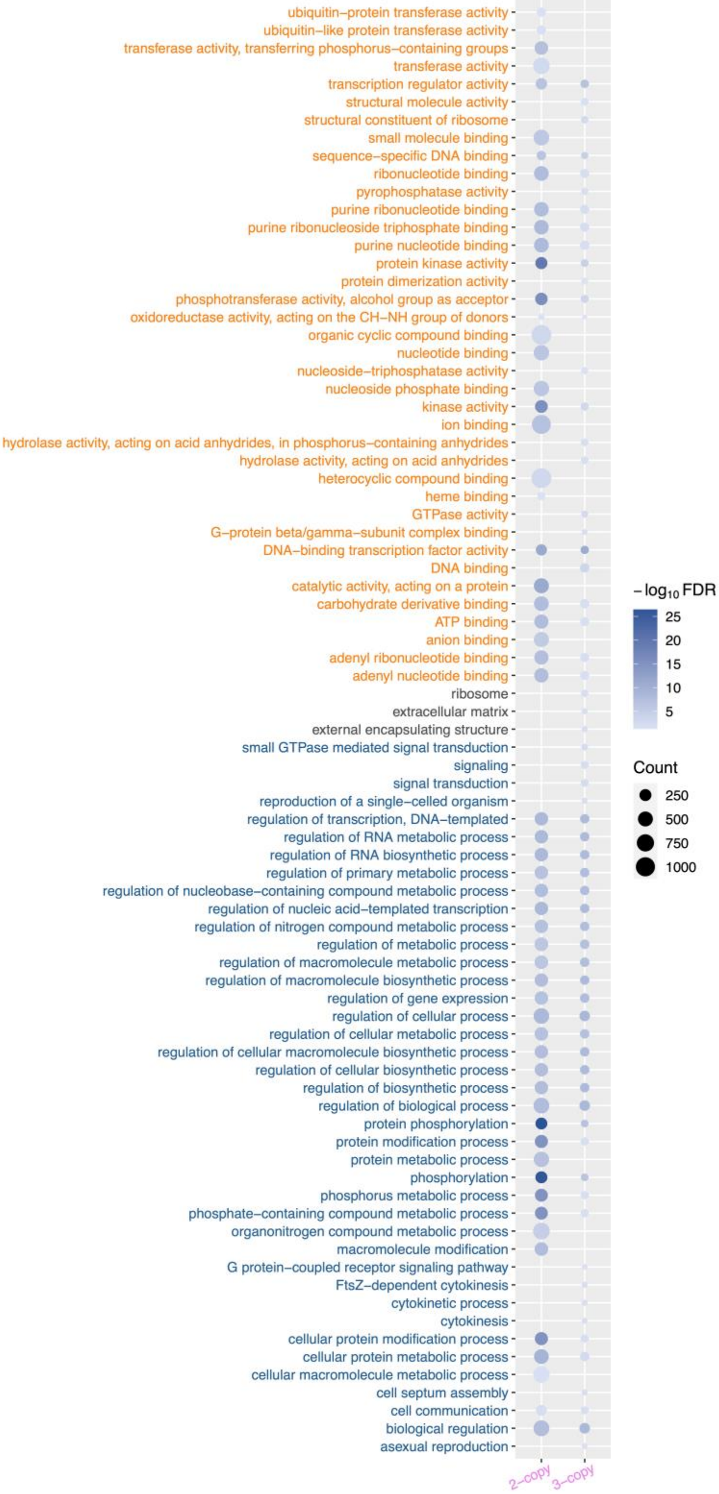

Supplementary Figure 32. Gene Ontology enrichment among the WGT retained gene duplicates compared with single genes in *L. speciosa*. The size and color of the bubbles represent the number of retention groups and FDR value. A darker bubble color on the scale indicates a smaller FDR value. The retention groups are shown on the *x*-axis and GO categories are shown on the *y*-axis. The orange, gray, and blue labels indicate molecular function, cellular component, and biological process. Source data are provided as a Source Data file.

Supplementary Table 1. Library and sequence statistics.

Species	Sequencing method	Number of reads	Total bases	Genome coverage
Sonneratia alba	Illumina paired-end reads	326,643,336[a]	32.99 Gb	161X
	PacBio SMRT long reads	3,456,716[a]	28.36 Gb	138X
	Hi-C reads	692,536,472	103.88 Gb	508X
Lagerstroemia speciosa	Illumina paired-end reads	274,410,258	41.16 Gb	128X
	PacBio SMRT long reads	9,566,630	95.60 Gb	299X
	Hi-C reads	361,254,098	54.19 Gb	169X

[a]Previously released Illumina paired-end and PacBio SMRT long reads [1].

Supplementary Table 2. BUSCO evaluation of genome assembly and annotation completeness.

	Sonneratia alba		*Lagerstroemia speciosa*	
	Genome	Protein	Genome	Protein
Complete BUSCOs (C)	2048 (96.6%)	1929 (90.9%)	2038 (96.1%)	1966 (92.7%)
Complete and single-copy BUSCOs (S)	1894 (89.3%)	1782 (84.0%)	1870 (88.2%)	1800 (84.9%)
Complete and duplicated BUSCOs (D)	154 (7.3%)	147 (6.9%)	168 (7.9%)	166 (7.8%)
Fragmented BUSCOs (F)	22 (1.0%)	69 (3.3%)	27 (1.3%)	76 (3.6%)
Missing BUSCOs (M)	51 (2.4%)	123 (5.8%)	56 (2.6%)	79 (3.7%)

Supplementary Table 3. Genome size and transposable element (TE) contents within each genome.

Order	Family	Species	Abbreviation	Assembled genome size (Mb)	TE length (Mb)	TE content (%)
Myrtales	Lythraceae	*Sonneratia alba*	sal	204.46	42.83	20.95
Myrtales	Lythraceae	*Lagerstroemia speciosa*	lsp	319.66	116.66	36.50
Myrtales	Lythraceae	*Punica granatum*	pgr	320.49	156.65	48.88
Myrtales	Myrtaceae	*Eucalyptus grandis*	egr	691.35	344.36	49.81
Vitales	Vitaceae	*Vitis vinifera*	vvi	486.20	254.68	52.38
Proteales	Nelumbonaceae	*Nelumbo nucifera*	nnu	821.29	507.79	61.83

Supplementary Table 4. The divergence times in this study and previously published studies.

Node	This study	TimeTree
Lagerstroemia-Punica	67.82 (61.73, 74.09)	54 (37.1, 70.6)
Punica-Eucalyptus	91.15 (81.56, 100.56)	85 (79.0, 91.1)
Eucalyptus-Arabidopsis	108.91 (99.75, 117.59)	110 (100.0, 112.2)
Arabidopsis-Vitis	122.52 (115.61, 128.62)	117 (109.8, 124.4)
Vitis-Nelumbo	125.72 (120.58, 129.14)	124 (121.5, 130.2)

*The numbers in parentheses indicate the range of the confidence interval.

Supplementary Table 5. RNA-seq data information of *S. alba*.

Sample	Number of reads	Total bases (Gb)
Leaf replicate 1	55,062,900	6.41
Leaf replicate 2	60,036,152	7.17
Leaf replicate 3	51,443,410	6.59
Root replicate 1	52,125,028	6.61
Root replicate 2	47,047,236	5.85
Root replicate 3	45,431,922	6.11
Flower replicate 1	55,188,814	6.40
Flower replicate 2	57,861,688	6.79
Flower replicate 3	49,714,586	6.23
Fruit replicate 1	50,517,482	6.42
Fruit replicate 2	47,846,372	6.10
Fruit replicate 3	47,491,936	6.09
Total	619,767,526	76.76

Supplementary Table 6. Summary of differentially expressed gene pairs (DEGPs) in *S. alba*.

Tissues	No. of DEGPs	Percentage of DEGPs	No. of two-copy groups with DEGPs	No. of three-copy groups with DEGPs
Flower	3270	63.69%	2105	516
Fruit	2980	58.04%	1937	481
Leaf	3315	64.57%	2136	504
Root	3171	61.76%	2058	495

Supplementary Table 7. RNA-seq data information of *Lagerstroemia speciosa*.

Sample	Number of reads	Total bases (Gb)
Leaf replicate 1	52,736,456	7.91
Leaf replicate 2	82,685,624	12.40
Stem replicate 1	69,440,234	10.42
Stem replicate 2	62,045,982	9.31
Flower replicate 1	59,375,332	8.91
Flower replicate 2	56,305,674	8.45
Fruit replicate 1	60,108,710	9.02
Fruit replicate 2	54,115,496	8.12
Total	496,813,508	74.52

Supplementary Table 8. Summary of differentially expressed gene pairs (DEGPs) in *L. speciosa*.

Tissues	No. of DEGPs	Percentage of DEGPs	No. of two-copy groups with DEGPs	No. of three-copy groups with DEGPs
Flower	4608	62.60%	2806	796
Fruit	4364	59.29%	2653	761
Leaf	4551	61.83%	2750	779
Stem	4583	62.26%	2776	794

Supplementary Table 9. The numbers of up-regulated genes across salinity conditions.

Conditions	Two-copy retention groups		Three-copy retention groups	
	No. of genes	No. of groups	No. of genes	No. of groups
Leaf (0mM *vs*. 250mM)	64	63	21	18
Leaf (250mM *vs*. 500mM)	298	281	103	83
Root (0mM *vs*. 250mM)	7	7	2	2
Root (250mM *vs*. 500mM)	4	4	0	0

*No. of groups: the number of the WGT retention groups with at least one up-regulated gene.

Supplementary Table 10. Statistics for *Lagerstroemia speciosa* genome with contig level.

Genome features	*Lagerstroemia speciosa* (Contig level assembly)
Sequencing methods	Illumina + PacBio
Estimated genome size	340.46 Mb (k-mer); 361 Mb (flow cytometry)
Assembled genome size	319.60 Mb
GC content	40.40%
Number of contigs	1273
N50 length	4.72 Mb
N90 length	208.40 Kb
Longest sequence length	8.67 Mb
Gap content	0%

Supplementary references

1. He, Z. *et al.* Convergent adaptation of the genomes of woody plants at the land–sea interface. *Natl Sci Rev* **7**, 978–993 (2020).
2. Li, H. & Durbin, R. Fast and accurate short read alignment with Burrows-Wheeler transform. *Bioinformatics* **25**, 1754–1760 (2009).
3. Li, H. Minimap2: pairwise alignment for nucleotide sequences. *Bioinformatics* **34**, 3094–3100 (2018).
4. Manni, M., Berkeley, M. R., Seppey, M., Simão, F. A. & Zdobnov, E. M. BUSCO update: novel and streamlined workflows along with broader and deeper phylogenetic coverage for scoring of eukaryotic, prokaryotic, and viral genomes. *Mol Biol Evol* **38**, 4647–4654 (2021).
5. Graham, S. A., Oginuma, K., Raven, P. H. & Tobe, H. Chromosome numbers in *Sonneratia* and *Duabanga* (Lythraceae s.l.) and their systematic significance. *Taxon* **42**, 35–41 (1993).
6. Rice, A. *et al.* The Chromosome Counts Database (CCDB) – a community resource of plant chromosome numbers. *New Phytologist* **206**, 19–26 (2015).
7. Wang, Y. *et al.* MCScanX: a toolkit for detection and evolutionary analysis of gene synteny and collinearity. *Nucleic Acids Res* **40**, e49 (2012).
8. Shannon, P. *et al.* Cytoscape: a software environment for integrated models of biomolecular interaction networks. *Genome Res* **13**, 2498–2504 (2003).
