# Supplementary material for: Genomic evidence for rediploidization and adaptive evolution following the whole-genome triplication
Source: Nat Commun. 2024 Feb 22;15:1635. doi: 10.1038/s41467-024-46080-7 (PMC10884412; doi:10.1038/s41467-024-46080-7)
Supplement: Supplementary file 3 — Description of Additional Supplementary Files [file 41467_2024_46080_MOESM3_ESM.pdf]

Description of Additional Supplementary Files

File Name: Supplementary Data 1
Description: Summary of karyotypes of typical mangrove species.

File Name: Supplementary Data 2
Description: The credits for the cartoon materials used in Fig. 3.

File Name: Supplementary Data 3
Description: Summary of GO categories enriched among differentially expressed gene pairs (DEGPs) in different tissues.

File Name: Supplementary Data 4
Description: Detailed annotation of three-copy retention groups related to root development and salt tolerance.

File Name: Supplementary Data 5
Description: Summary of whole genome sequences used in phylogenomic analysis in this study.
