# Supplementary material for: Genomic evidence for rediploidization and adaptive evolution following the whole-genome triplication
Source: Nat Commun. 2024 Feb 22;15:1635. doi: 10.1038/s41467-024-46080-7 (PMC10884412; doi:10.1038/s41467-024-46080-7)
Supplement: Supplementary file 9 — Reporting Summary [file 41467_2024_46080_MOESM9_ESM.pdf]

nature portfolio

Corresponding author(s): Ziwen He

Last updated by author(s): Feb 7, 2024

Reporting Summary

Nature Portfolio wishes to improve the reproducibility of the work that we publish. This form provides structure for consistency and transparency in reporting. For further information on Nature Portfolio policies, see our Editorial Policies and the Editorial Policy Checklist.

Statistics

For all statistical analyses, confirm that the following items are present in the figure legend, table legend, main text, or Methods section.

n/a	Confirmed	
☐	☒	The exact sample size (*n*) for each experimental group/condition, given as a discrete number and unit of measurement
☐	☒	A statement on whether measurements were taken from distinct samples or whether the same sample was measured repeatedly
☐	☒	The statistical test(s) used AND whether they are one- or two-sided<br>*Only common tests should be described solely by name; describe more complex techniques in the Methods section.*
☒	☐	A description of all covariates tested
☐	☒	A description of any assumptions or corrections, such as tests of normality and adjustment for multiple comparisons
☐	☒	A full description of the statistical parameters including central tendency (e.g. means) or other basic estimates (e.g. regression coefficient) AND variation (e.g. standard deviation) or associated estimates of uncertainty (e.g. confidence intervals)
☐	☒	For null hypothesis testing, the test statistic (e.g. F, t, r) with confidence intervals, effect sizes, degrees of freedom and P value noted<br>*Give P values as exact values whenever suitable.*
☐	☒	For Bayesian analysis, information on the choice of priors and Markov chain Monte Carlo settings
☐	☒	For hierarchical and complex designs, identification of the appropriate level for tests and full reporting of outcomes
☐	☒	Estimates of effect sizes (e.g. Cohen's *d*, Pearson's *r*), indicating how they were calculated

Our web collection on statistics for biologists contains articles on many of the points above.

Software and code

Policy information about availability of computer code

Data collection	PacBio SMRT reads were generated on the PacBio sequel II platform; Short-insert paired-end reads were generated on the Illumina HiSeq X Ten platform; RNA-seq reads were generated on the Illumina HiSeq X Ten or HiSeq 2500 or BGI-SEQ 500 platform; Hi-C data were generated on the Illumina NovaSeq 6000 or BGISEQ-500 platform.
Data analysis	Genome assembly, annotation and assessment: wtdbg2 (v2.5), Quiver (SMRT Analysis v2.3.0), Pilon (v1.22), HiC-Pro (v3.0.0), Juicer (v1.6), Juicebox (v1.13.01), 3D-DNA (v180922), RepeatMasker (open-4.0.9), RepeatModeler (v2.0), LTR_FINDER (v1.0.7), Augustus (v3.3.1), GeneMark (v2.5), exonerate (v2.2.0), Tophat2 (v2.1.1), Cufflinks (v2.2.1), EVidenceModeler (v1.1.1), iTAK (v1.7), BUSCO (v5);<br>Phylogenetic analysis: OrthoFinder (v2.4.0), MAFFT (v7.429), PAL2NAL (v14.0), Gblocks (v0.91b), RAxML-NG (v1.1.0), PAML (v4.9j), GGTREE (v2.2.4);<br>Whole-genome triplication and chromosome evolution analyses: BLASTP (v2.9.0+), MCScanX (v1), JCVI (v1), Circos (v0.69-7), KaKs_Calculator (v2.0), igraph (v1.5.0.1), RAxML-NG (v1.1.0), MAPS (v1), Notung (v2.9.1.5), WGDgc (v1.3), CAFE (v4.2.1), PAML (v4.9j), ChromEvol (v2.1), WGDI (v0.6.5);<br>Transcriptome analysis: SolexaQA++ (v3.1.7.1), HISAT2 (v2.2.0), HTSeq (v0.13.5), R (v3.4.4), Cytoscape (v3.7.2), DESeq2 (v1.40.2);<br>Selection analysis: BWA (v0.7.17-r1188), SAMtools (v1.9), Picard (v2.18.20), GATK (v3.7), VCFtools (v0.1.17), DEF-alpha (v2.16).

For manuscripts utilizing custom algorithms or software that are central to the research but not yet described in published literature, software must be made available to editors and reviewers. We strongly encourage code deposition in a community repository (e.g. GitHub). See the Nature Portfolio guidelines for submitting code & software for further information.

Data

Policy information about availability of data

All manuscripts must include a data availability statement. This statement should provide the following information, where applicable:

- Accession codes, unique identifiers, or web links for publicly available datasets
- A description of any restrictions on data availability
- For clinical datasets or third party data, please ensure that the statement adheres to our policy

The raw genomic Illumina reads, PacBio reads, Hi-C reads, and RNA-seq reads reported in this paper have been deposited in the Genome Sequence Archive (GSA, https://ngdc.cncb.ac.cn/gsa) in National Genomics Data Center, Beijing Institute of Genomics, Chinese Academy of Sciences / China National Center for Bioinformation, under accession number CRA004284 [https://ngdc.cncb.ac.cn/gsa/browse/CRA004284] with BioProject ID PRJCA005319 [https://ngdc.cncb.ac.cn/bioproject/browse/PRJCA005319]. The genome assembly sequences have been deposited in the Genome Warehouse (GWH, https://ngdc.cncb.ac.cn/gwh) in National Genomics Data Center under accession number GWHBCIQ00000000 [https://ngdc.cncb.ac.cn/gwh/Assembly/20653/show], GWHBCKL00000000 [https://ngdc.cncb.ac.cn/gwh/Assembly/20692/show] with BioProject ID PRJCA004930 [https://ngdc.cncb.ac.cn/bioproject/browse/PRJCA004930] and BioSample ID SAMC353197 [https://ngdc.cncb.ac.cn/biosample/browse/SAMC353197], SAMC353201 [https://ngdc.cncb.ac.cn/biosample/browse/SAMC353201]. The genome assemblies and annotations are also available at Figshare: Sonneratia alba [https://doi.org/10.6084/m9.figshare.25118819], Lagerstroemia speciosa [https://doi.org/10.6084/m9.figshare.25118831]. Source data are provided with this paper.

Research involving human participants, their data, or biological material

Policy information about studies with human participants or human data. See also policy information about sex, gender (identity/presentation), and sexual orientation and race, ethnicity and racism.

Reporting on sex and gender	N/A
Reporting on race, ethnicity, or other socially relevant groupings	N/A
Population characteristics	N/A
Recruitment	N/A
Ethics oversight	N/A

Note that full information on the approval of the study protocol must also be provided in the manuscript.

Field-specific reporting

Please select the one below that is the best fit for your research. If you are not sure, read the appropriate sections before making your selection.

☐ Life sciences ☐ Behavioural & social sciences ☒ Ecological, evolutionary & environmental sciences

For a reference copy of the document with all sections, see nature.com/documents/nr-reporting-summary-flat.pdf

Ecological, evolutionary & environmental sciences study design

All studies must disclose on these points even when the disclosure is negative.

Study description	We present two chromosome-scale genomes of Lythraceae plants: the mangrove tree Sonneratia alba and related inland plant Lagerstroemia speciosa , as a part of the worldwide mangrove genomes project. Through comprehensive analyses, we trace the evolutionary history of genomes, investigate the polyploidization–rediploidization process, and aim to elucidate adaptive evolution during global climate change. The genome sequences provided in this study will also expedite genomic and evolutionary research in plants.
Research sample	The mature specimens of Sonneratia alba and Lagerstroemia speciosa were sampled from the nursery of Dongzhai Harbor National Nature Reserve in Haikou and Sun Yat-sen University in Guangzhou. For resequencing data, 12 individuals of Sonneratia alba from Cebu, Philippines, and other 12 individuals from Davao, Philippines, were collected. All plants included in the study were adult individuals. Sampling within each population was conducted randomly to ensure the representativeness of the samples.
Sampling strategy	For de novo genome data, fresh and healthy tissues from a plant of Sonneratia alba and a plant of Lagerstroemia speciosa were harvested and immediately frozen in liquid nitrogen, respectively, followed by preservation at -80°C in the laboratory. For resequencing data, fresh and healthy leaves from 24 individuals of Sonneratia alba (12 individuals per population) were dried and preserved with silica gel. For RNA-seq data, fresh and healthy tissues from three individuals of Sonneratia alba and two individuals of Lagerstroemia speciosa were harvested and immediately frozen in liquid nitrogen, respectively, followed by preservation at -80°C in the laboratory. For population analysis, a sample size of 12 individuals is deemed sufficient; for expression analysis, biological replicates are provided in this study.
Data collection	Samples of Sonneratia alba from the nursery of Dongzhai Harbor National Nature Reserve in Haikou were collected by X.F., Q.C., G.L.,

	C.Z., S. Shi and Z.H. Samples of Sonneratia alba from Philippines were collected by S.X. and S. Shi. Samples of Lagerstroemia speciosa from Sun Yat-sen University in Guangzhou were collected by X.F., Q.C. and G.L. Plant samples information has been deposited in the BioSample database in National Genomics Data Center. High-throughput sequencing data were generated on different platforms, including PacBio, Illumina, and BGISEQ.
Timing and spatial scale	Spatial scale of sampling in this study is described in Methods section (including the nursery of Dongzhai Harbor National Nature Reserve in Haikou, Sun Yat-sen University in Guangzhou; Cebu and Davao in Philippines). All samples were collected between 2018-2023.
Data exclusions	No data were excluded.
Reproducibility	The leaf, root, flower, and fruit tissues of S. alba contains three independent biological replicates. The leaf, stem, flower, and fruit tissues of L. speciosa contains two independent biological replicates. And read coverages for each sample are from dozens to hundreds. All attempts at replication were successful.
Randomization	The mature plant samples for whole genome sequencing were randomly sampled with permission.
Blinding	Blinding is not applicable in this study as it does not involve subjects receiving different treatments.

Did the study involve field work? ☐ Yes ☒ No

Reporting for specific materials, systems and methods

We require information from authors about some types of materials, experimental systems and methods used in many studies. Here, indicate whether each material, system or method listed is relevant to your study. If you are not sure if a list item applies to your research, read the appropriate section before selecting a response.

Materials & experimental systems

n/a	Involved in the study	
☒	☐	Antibodies
☒	☐	Eukaryotic cell lines
☒	☐	Palaeontology and archaeology
☒	☐	Animals and other organisms
☒	☐	Clinical data
☒	☐	Dual use research of concern
☐	☒	Plants

Methods

n/a	Involved in the study	
☒	☐	ChIP-seq
☒	☐	Flow cytometry
☒	☐	MRI-based neuroimaging

Dual use research of concern

Policy information about dual use research of concern

Hazards

Could the accidental, deliberate or reckless misuse of agents or technologies generated in the work, or the application of information presented in the manuscript, pose a threat to:

No	Yes	
☒	☐	Public health
☒	☐	National security
☒	☐	Crops and/or livestock
☒	☐	Ecosystems
☒	☐	Any other significant area

Experiments of concern

Does the work involve any of these experiments of concern:

No	Yes	
☒	☐	Demonstrate how to render a vaccine ineffective
☒	☐	Confer resistance to therapeutically useful antibiotics or antiviral agents
☒	☐	Enhance the virulence of a pathogen or render a nonpathogen virulent
☒	☐	Increase transmissibility of a pathogen
☒	☐	Alter the host range of a pathogen
☒	☐	Enable evasion of diagnostic/detection modalities
☒	☐	Enable the weaponization of a biological agent or toxin
☒	☐	Any other potentially harmful combination of experiments and agents
